# Evaluation of *Moringa oleifera* extract and metal-organic frameworks (MOFs) as therapeutic agents against chronic *Toxoplasma gondii* infection in mice

**DOI:** 10.1038/s41598-026-64672-9

**Published:** 2026-08-05

**Authors:** Ashraf M. Barakat, Doaa Amer, Fatma El-Lessy, Mona Elderbawy, Rehab Mohamed El Shahat, Sabry A. S. Sadek, Reda M. Abdelhameed

**Affiliations:** 1 Department of Zoonotic Diseases, Veterinary Research Institute, National Research Centre, El Buhouth Street, Dokki, Post Box 12622, Cairo, Egypt; 2 Department of Parasitology, Faculty of Medicine, Al-Azhar University, Cairo, Egypt; 3 Department of Pharmacology, Faculty of Medicine for Girls, Al-Azhar University, Cairo, Egypt; 4 Applied Organic Chemistry Department, Chemical Industries Research Institute, National Research Centre, Dokki, Giza, Egypt

**Keywords:** Toxoplasmosis, *Moringa Olifera*, Cu-MOFs, RT-PCR, Treatment, Histopathology, Biotechnology, Diseases, Drug discovery, Immunology, Microbiology

## Abstract

Toxoplasmosis, a zoonotic infection caused by the protozoan *Toxoplasma gondii*, remains a significant global health concern, with seroprevalence exceeding 60% in specific regions. Given the limitations of current therapies for chronic stages, this study investigated the therapeutic efficacy of *Moringa oleifera* (MO) extract and MO-loaded copper-based metal-organic frameworks (MO@L-AA-Cu) in a murine model. Sixty-five female Swiss albino mice were utilized. Following the establishment of a negative control group (n=5), the remaining mice were orally inoculated with 20 cysts of the *T. gondii* ME49 strain to induce chronic infection. Treatment efficacy was evaluated via brain cyst quantification, histopathological examination of the brain, eyes, and spleen, and molecular analysis using RT-PCR. All treatment groups exhibited significant reductions in parasitic load compared to the positive control. The highest reduction was observed in group G3e (73.61%), followed by G3c (61.64%), G3d (56.91%), G3a (54.64%), and G3b (44.33%). Histopathological findings demonstrated a marked attenuation of inflammatory infiltrates, fibrosis, and structural tissue damage in groups receiving MO and Spiramycin encapsulated within Cu-MOFs. RT-PCR results corroborated these findings, identifying the lowest parasite DNA levels in the G3e cohort. The integration of MO extract and Spiramycin into L-AA-Cu MOFs significantly enhances their antiparasitic and anti-inflammatory properties. These findings suggest that MO-loaded MOFs represent a promising nanotherapeutic platform for the treatment of chronic toxoplasmosis, offering superior efficacy over conventional formulations.

## Introduction

*Toxoplasma gondii* (T. gondii) is a globally occurring parasite that depends on intracellular habitation^1,2^. T. gondii is responsible for toxoplasmosis in humans and is primarily a zoonotic pathogen, capable of being transmitted between animals and humans^3^. Toxoplasma infection affects over 60% of individuals in certain populations, with the highest prevalence observed in regions with warm, humid climates—conditions that favor the long-term survival of oocysts. According to the Centers for Disease Control and Prevention (CDC), toxoplasmosis is not typically transmitted between individuals, except in specific cases such as congenital transmission (from mother to fetus), blood transfusions, and organ transplants. The primary modes of human infection include three major routes: ingestion of undercooked contaminated meat, consumption of food or water contaminated with oocysts and vertical transmission during pregnancy^4^. In people with normal immunity, the infection usually develops with mild or no symptoms. In individuals with weakened immune systems, toxoplasmosis can result in serious or potentially life-threatening complications. The standard therapy combination of pyrimethamine and sulfadiazine often proves ineffective in chronic infections and is associated with various adverse effects.

In search of alternative treatments, the antiparasitic properties and potential mode of action of an aqueous extract from MO seeds were examined in vitro. Cytotoxicity of the MO extract on HeLa cells was assessed using an MTT assay (3-(4,5-dimethylthiazol-2-yl)-2,5-diphenyltetrazolium bromide). The results indicated that concentrations below 50 μg/mL were non-toxic to HeLa cells. The MO extract inhibited T. gondii invasion and intracellular replication, with results similar to the effect of Sulfadiazine with Pyrimethamine, and demonstrated cellular nitric oxide production at a concentration of 30 μg mL^−1^^5^. MO oil encapsulated in solid lipid nanoparticles (SLNs) exhibits potent anti-Toxoplasma activity by effectively eliminating T. gondii tachyzoites while maintaining low cytotoxicity. These nano-emulsions, particularly when combined with conventional therapeutic agents, hold promise as an effective strategy for the treatment of toxoplasmosis^6^. The use of MO reduced 95.8% quantity of T. gondii oocysts^7^.

Sulfadiazine and Pyrimethamine are commonly prescribed drugs for treating human toxoplasmosis. However, despite their effectiveness against the acute phase, they do not eliminate chronic infection and are associated with a range of adverse effects, including hematological toxicity, hypersensitivity reactions, gastrointestinal issues, teratogenicity, intolerance, and bone marrow suppression^8^. The therapeutic and preventive approaches for T. gondii infection rely on extracts and/or compounds derived from natural products, which have proven effective as alternative treatment options over the last 20 years. An ethanolic extract of ginger was used to prepare zinc oxide nanoparticles (ZnO NPs). The effects of treatment on histopathological changes accompanied by toxoplasmosis were studied^9^. Copper-based nanomaterial was evaluated for antioxidant, antibacterial, and anticancer/cytotoxic activity- properties parallel to those assessed for the Cu–MOF platform^10^. The immunological and histopathological evaluation framework in an infection model was parallels previous work^11^.

In recent years, ethnopharmacological approaches have gained traction, with MO emerging as a candidate of particular interest. Known as the "miracle tree," MO possesses a rich profile of bioactive secondary metabolites, including flavonoids, phenolic acids, and isothiocyanates, which exhibit potent antimicrobial, anti-inflammatory, and antioxidant properties. However, the clinical application of crude botanical extracts is often hindered by poor bioavailability, rapid metabolic degradation, and challenges in crossing biological barriers, such as the blood-brain barrier (BBB), which is critical for treating cerebral toxoplasmosis^12^.

To overcome these pharmacokinetic limitations, nanotechnology offers a transformative solution through the use of MOFs, it is a class of crystalline porous materials composed of metal ions or clusters coordinated to organic linkers^13–19^. Their exceptionally high surface area, tunable pore size, and high loading capacity make them superior delivery vehicles compared to traditional lipid or polymer-based nanoparticles. Specifically, copper-based MOFs (Cu-MOFs) have garnered attention due to the inherent biocidal properties of copper ions, which may act synergistically with encapsulated therapeutic agents^20^. By utilizing MOFs as a delivery platform, bioactive compounds can be protected from premature degradation and delivered with greater precision to infected tissues. Recently, chronic toxoplasmosis was treated using curcumin and amino-functionalized MOFs derived from Fe-MOFs and UiO-66-NH_2_^21^. Additionally, black seed and wheat germ oils were encapsulated within MOFs to tackle chronic murine toxoplasmosis^22^. Moreover, MOF nanoparticles were loaded with honeybee venom to address chronic toxoplasmosis^23^. Unlike previously reported nano-formulations that primarily rely on the MOF as a passive, biocompatible delivery vehicle for a single isolated bioactive molecule, our approach leverages a strategic dual-action mechanism. By utilizing a copper-based MOF (Cu-MOF) as the host matrix for MO extract, we exploit the intrinsic, documented anti-parasitic properties of copper ions in synergetic combination with the multi-targeted polyphenolic and flavonoid profile of *Moringa oleifera*. Furthermore, chronic toxoplasmosis therapy is severely limited by the inability of conventional drugs to effectively penetrate the thick cyst wall of *Toxoplasma gondii* bradyzoites within host tissues. A comprehensive comparison between MO@L-AA-Cu and previously reported MOF systems is summarized in Table 1.

**Table 1:** Comparative Analysis of MO@L-AA-Cu vs. Reported MOF Platforms for treatment of Toxoplasma

MOFs	Mechanistic pathway	Release behavior	Biocompatibility	Key innovation	References
Fe-based MOFs	Fenton-like reaction (ROS generation)	Frequent burst release; depends on physical adsorption	Moderate; potential iron accumulation risks	Established Fenton chemistry	^21^
Zr-based MOFs	High thermal/hydrolytic stability, passive carrier	Requires surface modification or linkers to prevent premature leakage	Good chemical stability, but slow clearance *in vivo*	High structural stability	^21^
Natural product load-MOFs	Pharmacological activity derived primarily from loaded natural products	Dependent on non-covalent encapsulation stability	Depends heavily on natural product load dosage	Bioactive natural product delivery	^22,23^
MO@L-AA-Cu	Synergistic multi-modal activity (L-AA-mediated Cu^+^/Cu^2+^ redox cycling + targeted ROS generation)	Enhanced coordination/trapping via L-AA modification, preventing premature leakage	High inherent biocompatibility (uses endogenous /nutrient-derived L-AA)	Intrinsically active, biomolecule-integrated catalytic framework offering targeted, highly efficient therapy	Current work

This work fills a critical knowledge gap by demonstrating how the unique surface topology and controlled release kinetics of this specific Cu-MOF architecture enhance the bioavailability and targeted delivery of the crude extract, achieving superior cysticidal efficacy and reduced tissue pathology compared to previously documented mono-therapeutic nano-systems. This study aims to evaluate the therapeutic synergy between MO extract, Spiramycin, and Cu-based MOFs against chronic *T. gondii* (ME49 strain) infection. By leveraging the high loading efficiency of MOFs, we hypothesize that this nanotherapeutic approach will significantly reduce parasitic burden and attenuate tissue-specific inflammation compared to conventional treatment modalities.

Although coordination frameworks and metal-organic networks have emerged as powerful platforms for encapsulation, conventional systems frequently rely on rigid, synthetic organic ligands (such as terephthalate or imidazole derivatives) that present long-term toxicity, low bio-renewability, and poor environmental compatibility. To circumvent these issues, recent research has shifted toward biomolecule-based frameworks (bio-MOFs) utilizing endogenous ligands like amino acids or vitamins (L-ascorbic acid). However, a critical knowledge gap remains unresolved in two main areas. Pure vitamin-derived metal complexes, such as pristine L-ascorbic acid-copper networks (L-AA-Cu), often suffer from high structural vulnerability in aqueous media, leading to premature matrix dissolution and an uncontrolled burst release of guest molecules. While plant extracts like *Moringa oleifera* possess exceptional multi-functional bioactivities (antioxidant, antimicrobial, and biostimulant properties), directly encapsulating such chemically diverse, multi-component phytochemical mixtures into stable nano-carriers without causing phase separation or loss of bioactivity remains a significant challenge. The precise interfacial chemistry and coordination mechanisms driving the stabilization of complex herbal extracts within an endogenous vitamin-metal network have not yet been systematically established. To address these limitations, this study introduces a synergistic strategy by developing a MO@L-AA-Cu composite. By using the multi-functional phenolic and flavonoid components of *Moringa oleifera* extract as both natural capping agents and auxiliary stabilizing ligands, we successfully overcome the rapid degradation typical of pristine ascorbic acid-copper networks. This approach creates a highly stable, eco-friendly composite that achieves a smart, pH-responsive, anomalous transport release mechanism.

## Experimental

### Chemicals

Copper nitrate hexahydrate (Cu(NO_3_)_2_·6H_2_O, 98%, Fluka), sodium hydroxide (NaOH, 99%, Merck), and L(+)-aspartic acid (L-AA, 98%, Acros). This study was performed at the National Research Centre (NRC) and the Faculty of Medicine, Al-Azhar University, from June to July 2024. The experiment adhered to internationally recognized guidelines and was approved by the Research Ethics Committee of the National Research Centre (NRC), Egypt.

*Moringa oleifera* plant material was cultivated in National Research Centre, Egypt, and Prof. Dr. Said A. Saleh, Chairman of Egyptian Scientific Association of Moringa, National Research Centre, Egypt provided us oil extract of *Moringa oleifera*. The seed of *Moringa oleifera* was collected from Agriculture Garden the Egyptian Scientific Society of Moringa (ESSM), National Research Centre, Dokki, Cairo, Egypt with voucher number M287.

### Synthesis of L-AA-Cu MOFs

Here is how to prepare MOFs based on L-AA in an aqueous medium: 50 mL of double-distilled water (DDW) was mixed with 1.0 g of L-AA in a 100 mL beaker and stirred at 500 rpm. To facilitate dissolution, 1 M sodium hydroxide (NaOH) was added intermittently. Once fully dissolved, the solution’s pH was adjusted to 7.0 using 1 M HCl. Stoichiometric amounts of aqueous Cu (II) nitrate solution were then added dropwise to the L-AA solution at pH 7 under continuous stirring at 750 rpm for 12 hours. During the reaction, a blue-colored solid (L-AA-based Cu (II) MOFs) precipitated, which was subsequently filtered and washed sequentially with water, a 1:1 (v/v) water-ethanol mixture, and water. Finally, the product was lyophilized using a freeze-dryer and stored in sealed tubes for further use.

### Moringaloading [MO@L-AA-Cusynthesis] and release

Moringa oil was diluted in 100 ml of ethanol at different concentrations (100–1,000 ppm) to load into L-AA-Cu MOF nanoparticles. A magnetic stirrer was then used to add 1 gram of L-AA-Cu MOF nanoparticles to the Moringa oil solution and stirred for 90 minutes at room temperature at 600 rpm. The mixture was then left alone overnight. The upper liquid was separated from the precipitate by centrifuging the resulting suspension for five minutes at 5,000 rpm. The difference between the pre-and post-loading Moringa oil concentrations was used to determine the amount of oil loaded. The percentage Loading Efficiency (LE%) was calculated in triplicate (n = 3) using the following equation:

$${\mathrm{Moringa}}\,{\mathrm{oil}}\,{\mathrm{loading}}{\mkern 1mu} \left( \% \right) = \left[ {\left( {{\mathrm{A}} - {\mathrm{B}}} \right)/{\mathrm{A}}} \right] \times 100$$

where A and B represent the initial and final MO concentration of the Moringa solution

The encapsulation of Spiramycin and MO extract within the L-AA-Cu framework was quantified indirectly by analyzing the supernatant after centrifugation using UV-Vis spectrophotometry at the maximum absorption wavelengths (λ_max_= 232nm for Spiramycin and 325nm for MO polyphenols).Calibration curves were established using five standard concentrations in triplicate, yielding highly linear relationships. For spiramycin the calibration equation was y = 0.024x + 0.015 (R^2^ = 0.998; linear range: 5-50 µg/mL; LOD = 0.85 µg/mL; LOQ = 2.58 µg/mL). For MO the calibration equation was y = 0.031x + 0.008 (R^2^ = 0.997; linear range: 10-100 µg/mL; LOD=1.20 µg/mL; LOQ = 3.64 µg/mL).

In vitro release studies were performed in triplicate (n = 3) at 37ºC under continuous shaking (100 rpm) in two distinct release media to evaluate pH-dependent behavior. To mimic normal physiological conditions and systemic blood circulation, phosphate-buffered saline (PBS, pH 7.4) was used. To simulate the acidic intracellular environment of host cell lysosomes/endosomes and the parasitophorous vacuole of *T. gondii* during infection, acetate buffer (pH 5.5) was used.At predetermined time intervals, aliquots of 1mL were withdrawn, centrifuged, and analyzed spectrophotometrically, with an equal volume of fresh buffer added to maintain sink conditions.

### Characterization of L-AA-Cu MOF and MO@L-AA-Cu

Using X-ray diffraction (XRD) patterns (Philips MPD X-ray diffractometer), the phase purity and crystallinity of the produced materials were evaluated; monochromatic copper (Cu Kα) was used. Using a scanning electron microscope (SEM: FEI Quanta FEG 250), the nanostructure morphology of the MOFs was examined. The elemental composition, localized purity, and elemental distribution profiles of the pristine framework and the encapsulated nanocomposite were evaluated using Energy-Dispersive X-ray (EDX) spectroscopy attached to a FEI Quanta FEG 250 Scanning Electron Microscope. The dry powder samples were dispersed on double-sided conductive carbon tape mounted on aluminum stubs and sputter-coated with a thin, conductive layer of gold under vacuum. EDX spectra were acquired at an accelerating voltage of 20 kV to record the relative weight and atomic percentages of Carbon (C), Oxygen (O), Nitrogen (N), and Copper (Cu). The surface chemical functional groups and coordination modes of the pristine components and the hybrid nanocomposites were investigated using a Bruker Vertex 70Fourier-Transform Infrared (FTIR) spectrophotometer. The spectra were recorded in the wavenumber range of 4000–500 cm^−1^ with a resolution of 4 cm^−1^. The solid samples were finely ground with spectroscopic-grade Potassium Bromide (KBr) and pressed into translucent pellets prior to analysis under ambient conditions. The textural characteristics, specific surface areas, and porosity profiles of the synthesized materials were evaluated via nitrogen (N_2_) adsorption-desorption isotherms at 77K using Quantachrome Nova Touch surface area and pore size analyzer. Prior to the measurements, the samples were subjected to an optimized degasification process under vacuum at 100°C 6 hours to thoroughly eliminate any moisture and coordinated guest solvents from the pore channels without collapsing the framework architecture. Hydrodynamic diameter, particle size distribution, and surface zeta potential of the nanocomposite were determined using a Malvern Zetasizer Nano ZS at 25 °C. Samples were dispersed in ultrapure water (0.1 mg/mL) and sonicated for 10 minutes prior to measurement. To evaluate colloidal stability under physiological conditions, the nanocomposites were incubated in phosphate-buffered saline (PBS, pH 7.4) and PBS supplemented with 10% fetal bovine serum (FBS) at 37 °C with gentle agitation. The hydrodynamic size and polydispersity index (PDI) were monitored over a period of 72 h at pre-determined time intervals (0, 12, 24, 48, and 72 h).

### Parasite strain

A virulent strain of *T. gondii* [ME49] was obtained from the Zoonotic Diseases Department (NRC), Egypt. The mouse passages were maintained every eight weeks to obtain fresh cysts. Cysts taken from mouse brains were used for infection. They were diluted with 1 mL of saline solution and used to infect mice at a dose of 20 cysts per mouse.

### Animals and experimental design

A total of 75 laboratory-bred female Swiss Albino mice, 6-8 weeks old and weighing about 20-25g, were purchased from the animal house, National Research Center, Cairo, Egypt, andwere divided into seven groups. Ten mice per group, except for the normal control group, which included five mice. Additionally, five uninfected mice were treated with silver nanoparticles (0.72 mg/kg) to assess histopathological changes in the liver. Treatments were administered orally via an esophageal tube starting on the first day of infection. All mice were sacrificed on the sixty-day post-infection for analysis. Mice were orally administered 20 *T. gondii* cysts per mouse and divided into seven groups (10 mice per group), except for the normal control group, which included five mice. An additional infected, non-treated group was also included. The remaining infected groups received treatment with MO and Spiramycin, either alone or loaded with L-AA-Cu MOFs, to evaluate histopathological changes in the brain, eyes, liver, and spleen. Treatments were given orally through an esophageal tube for 15 days, starting 45 days post-infection. All mice were sacrificed on the sixth day post-treatment for analysis. The experimental groups were categorized as follows:

1. Group 1: 5 mice were the normal control, consisting of no infection, no treatment mice.
2. Group 2: 10 mice were the infected mice with no treatment as a control.
3. Group 3: 50 mice were subdivided equally as 10mice in each subgroupas follow:
  - This subgroup received L-AA-Cu MOF at a dose of 100 mg/kg/day^9^.
  - This subgroup was treated with Spiramycin alone at a dose of 200 mg/kg for two weeks.
  - This subgroup received Spiramycin@L-AA-Cu.
  - This subgroup received *Moringa extract* alone at a dose of 0.2 mg/kg for 7 days^5^.
  - This subgroup received MO@L-AA-Cu at a 200 mg/kg dose for 1 week.

**Fig. 1:**
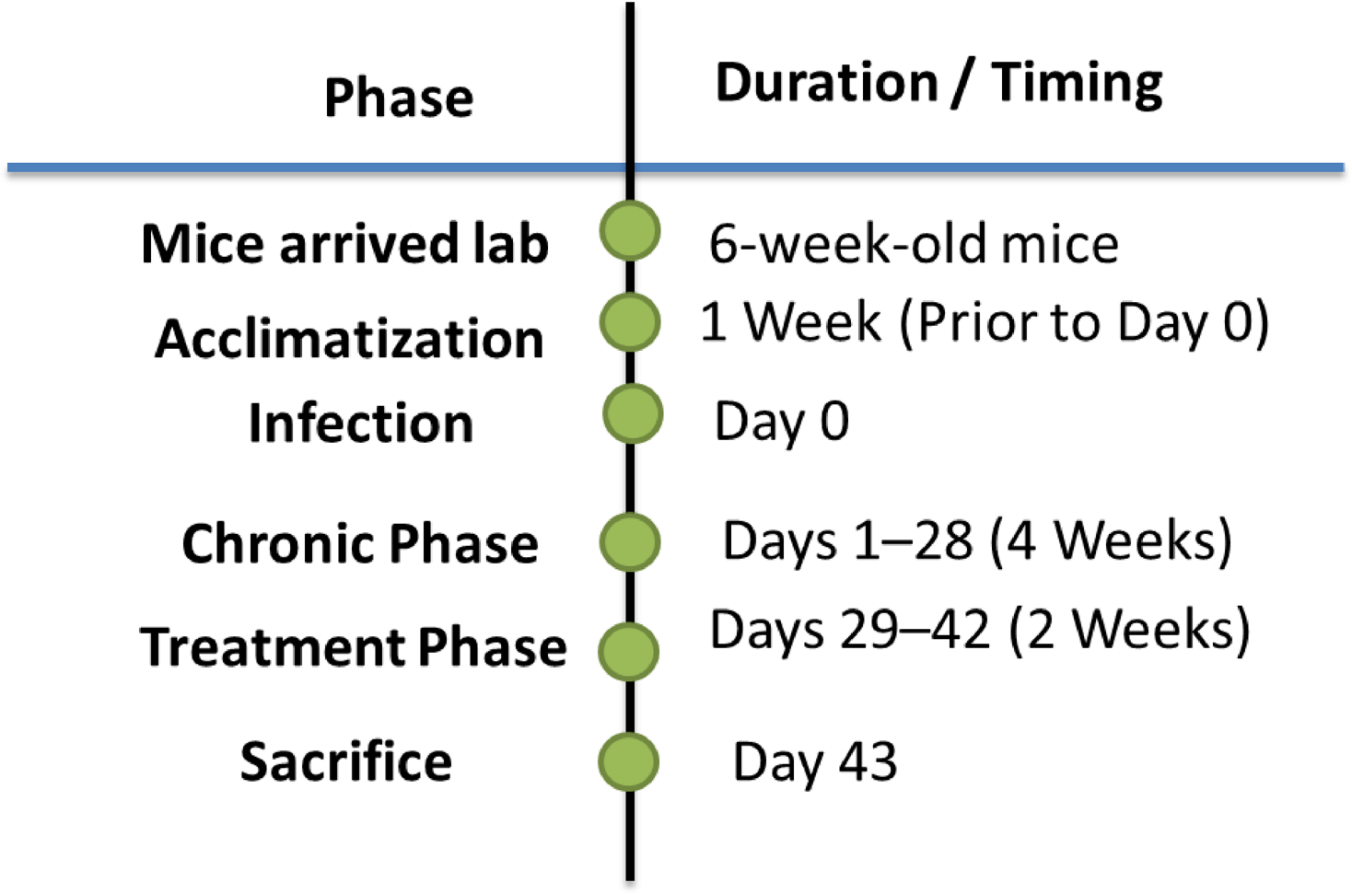
Timeline of the experimental protocol explaining the age of mice in weeks, treatments, and sampling

**Fig. 2:**
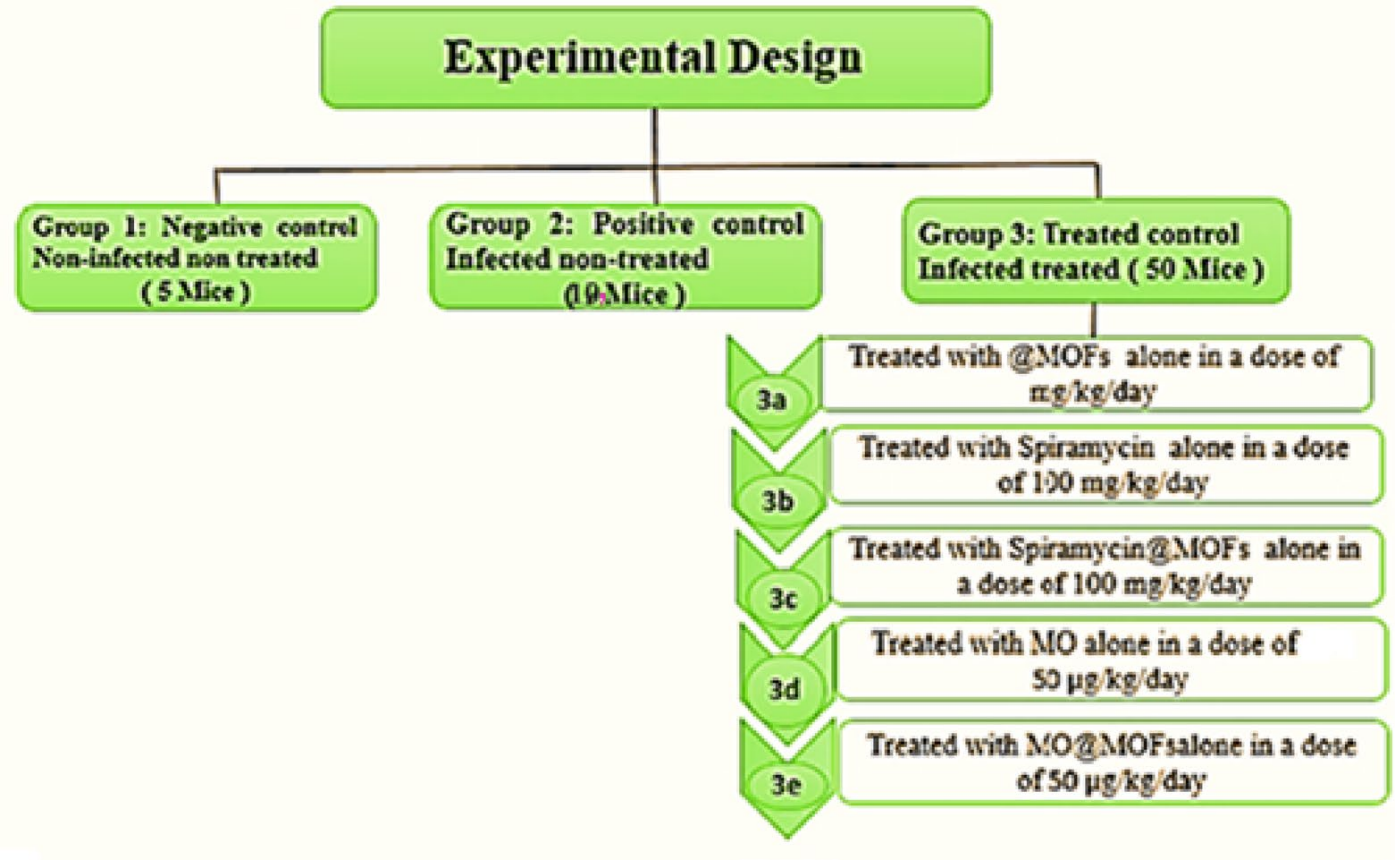
Schematic representation of the experimental design and chronicity induction protocol. Swiss albino mice (n=70) were partitioned into six experimental groups. Chronic toxoplasmosis was induced via oral gavage of 20 *Toxoplasma gondii* ME49 cysts. The timeline illustrates the infection phase, the subsequent administration of MO and MO@L-AA-Cu, and the final 60-day endpoint for parasitic load quantification (brain cyst count), histopathological assessment, and RT-PCR analysis.

Figure 1 Schematic diagram illustrating the experimental design and chronological timeline of the study. Following a 1-week acclimatization phase, mice were infected on Day 0 with the *T. gondii* ME49 strain. The chronic infection model was established over a 4-week period (Days 1–28), followed by a 2-week daily therapeutic regimen (Days 29–42). Ethical sacrifice and downstream tissue harvesting were executed on Day 43. Fig. 2 showed experimental design and infection schedule.

### Parasite cysts counting

After sacrificing the mice was done under anesthesia Ketamine (80 mg/kg IP), the abdominal cavity was opened, and the brain from each mouse in each group was collected for analysis. A 20 μL sample was placed on glass slides, which were then left to dry completely in the air at room temperature. Once dried, the slides were fixed in absolute methanol and subsequently stained with Giemsa stain for one minute. After staining, the slides were rinsed with tap water and examined under a bright-field microscope at 40 × magnification to count tachyzoites in the 20 μl sample. The tachyzoite count was then extrapolated to estimate the number per milliliter^24^.

Brain harvesting cysts (chronic) samples were counted directly in an average of 10 mg by a hemocytometer. The brain tissue was homogenized with an equal volume of PBS, pH 7.4, and passed through a 16-gauge needle ten times using a syringe to release tissue cysts^25^. The Average Parasite Load (APL/10 mg tissue) was measured across visceral organs and brain tissue during the acute and chronic treatment phases. Parasite load was assessed by quantifying the total number of free bradyzoites and within tissue cysts, per milligram of brain tissue, following the method described by El Fadaly et al.^26^. One drop of brain homogenate was placed on a slide, and under a blood cell counter, the average of every 10 mg was counted under a microscope.

### Cytotoxic effect on human normal fibroblast cell line (BJ1)

Cell viability was assessed by the mitochondrial dependent reduction of yellow MTT (3-(4,5-dimethylthiazol-2-yl)-2,5-diphenyl tetrazolium bromide) to purple formazan^27^. Procedure: All the following procedures were done in a sterile area using a Laminar flow cabinet biosafety class II level (Baker, SG403INT, Sanford, ME, USA). Cells were suspended in DMEM-F12 medium, 1% antibiotic-antimycotic mixture (10,000U/ml Potassium Penicillin, 10,000 µg/ml Streptomycin Sulfate and 25 µg/mL Amphotericin B) and 1% L-glutamine at 37 ºC under 5% CO_2_. Cells were batch cultured. The absorbance was then measured using a microplate multi-well reader (Bio-Rad Laboratories Inc., model 3350, Hercules, California, USA) at 595 nm and a reference wavelength of 620 nm. A statistical significance was tested between samples and negative control (cells with vehicle) using independent t-test by SPSS 11 program. DMSO is the vehicle used for dissolution of plant extracts and its final concentration on the cells was less than 0.2%. The percentage of change in viability was calculated according to the formula:

$$\left( {\left( {{\mathrm{Reading}}\,{\mathrm{of}}\,{\mathrm{extract}}/{\mathrm{Reading}}\,{\mathrm{of}}\,{\mathrm{negative}}\,{\mathrm{control}}} \right) - 1} \right) \times 100$$

A probit analysis was carried for IC50 and IC90 determination using SPSS 11 program.

### Histopathological analysis and cytotoxic activity

Liver tissue specimens from each mouse were individually fixed in 10% neutral buffered formalin, dehydrated using graded alcohol concentrations, cleared with xylene, and embedded in paraffin blocks. Tissue sections, 5 µm thick, were stained with H&E for histopathological analysis^28,29^. The cytotoxic effect on the human normal fibroblast cell line (BJ1) was assessed using the MTT assay. Cell viability was determined by the mitochondria-dependent reduction of MTT yellow (3-(4,5-dimethylthiazol-2-yl)-2,5-diphenyltetrazolium bromide) to purple formazan^27^.

### Histopathological evaluation and semiquantitative scoring

To eliminate observer bias, a blinded scoring procedure was implemented. All tissue slides were assigned randomized codes by an independent researcher, ensuring the investigating pathologist was completely blinded to the experimental treatment groups. Semiquantitative histopathological grading was performed across multiple randomly selected high-power fields (HPFs, X400) per animal. Inflammatory cell infiltration, tissue necrosis, and structural degeneration were scored using a standardized scale from (−) to (+++) adapted from validated inflammatory scoring systems. Negative (−), mild (+), moderate (++) and severe (+++).

### Quantitative real-time PCR (qPCR) for *Toxoplasma gondii* burden

Murine brain tissue specimens (T_1_–T_6_, 20–25 mg each) and parallel whole blood samples (B_1_–B_6_, 200 µL each, collected in EDTA-coated tubes) were obtained from six experimental mice per group. Total genomic DNA was isolated using the MagMAX™ CORE Nucleic Acid Purification Kit (Cat. No. A32700, Thermo Fisher Scientific, Waltham, MA, USA) in strict accordance with the manufacturer’s operational protocol. Purified DNA was eluted in 50 µL of elution buffer, and nucleic acid concentration and purity were verified spectrophotometrically (A_260_/A_280_ ratio > 1.8). Quantitative real-time PCR (qPCR) assays were performed to evaluate parasite burden targeting the highly conserved *T. gondii* P29 gene. Amplification was conducted using the ViPrimePLUSTaq qPCR Green Master Mix I (SYBR® Green Dye, Cat. No. QLMM12, Vivantis Technologies, Malaysia). Each 20 µL reaction mixture comprised:

- 2 µL of template DNA,
- 10 µLof 2X Green Master Mix,
- 0.1 µL (50 nmol) of each forward and reverse primer, and
- 7.8 µL of nuclease-free water.

Target-specific primers were meticulously designed using Lasergene DNASTAR software (DNASTAR, Madison, WI, USA) and are listed in Table 2. To normalize target gene expression and account for variations in DNA loading, murinewas utilized as the endogenous reference gene, evaluated under identical cycling conditions. Thermal cycling was executed on a qTOWER^3^ G thermal cycler (Analytik Jena, Germany) utilizing the following profile:

**Table 2:** Sequence of the P29 gene oligonucleotide primer

Primer name	Primer sequences
P29 Q-f	CAGCATGGATAAGGCATCTG
P29 Q-r	GTTGCTCCTCTGTTAGTTCC

Initial Denaturation: 95°C for 5 min. **A**mplification (40 cycles): Denaturation at 95°C for 30 s, followed by simultaneous annealing and extension at 60°C for 1 min.

Fluorescence data collection was captured at the immediate conclusion of each annealing/extension phase. All biological samples and negative controls (NTC) were processed in technical triplicates to ensure reproducibility. To confirm amplification specificity and verify the absence of primer-dimers or artifacts, a post-amplification dissociation (melt) curve analysis was systematically conducted by continuously measuring fluorescence during heating from 60°C to 95°C. Primer amplification efficiency (E) was verified via a 5-fold serial dilution standard curve, yielding an efficiency of 97.4%. Relative quantification of parasite DNA load was calculated using the comparative threshold cycle normalization method. Samples exhibiting no amplification profile up to cycle 40 were designated as below the assay’s analytical limit of detection (LoD).

### Statistical analysis

Experimental data are expressed as the mean ± standard deviation (SD) from at least three independent biological replicates. Prior to parametric evaluation, the assumption of normality was rigorously assessed using the Shapiro-Wilk test, and the homogeneity of variances was verified via Levene’s test. Statistical significance across multiple experimental groups was determined using one-way Analysis of Variance (ANOVA), followed by Tukey’s Honestly Significant Difference (HSD) post-hoc test for multiple pairwise comparisons. In cases where unequal variances were encountered, Welch’s ANOVA combined with Games-Howell post-hoc analysis was applied. Statistical reporting includes the F-statistic, degrees of freedom (df), 95% confidence intervals (95% CI), and effect sizes. To quantify the magnitude of the experimental effects, effect sizes were calculated using partial eta-squared for ANOVA models. 95% confidence intervals (95% CI) were determined for primary outcome metrics. All statistical computations were executed using GraphPad Prism version 9.0 / IBM SPSS Statistics version 26.0 / R software. A *p*-value of < 0.001 was considered statistically significant.

## Results

### Characterization of the nanocomposites

**Fig. 3:**
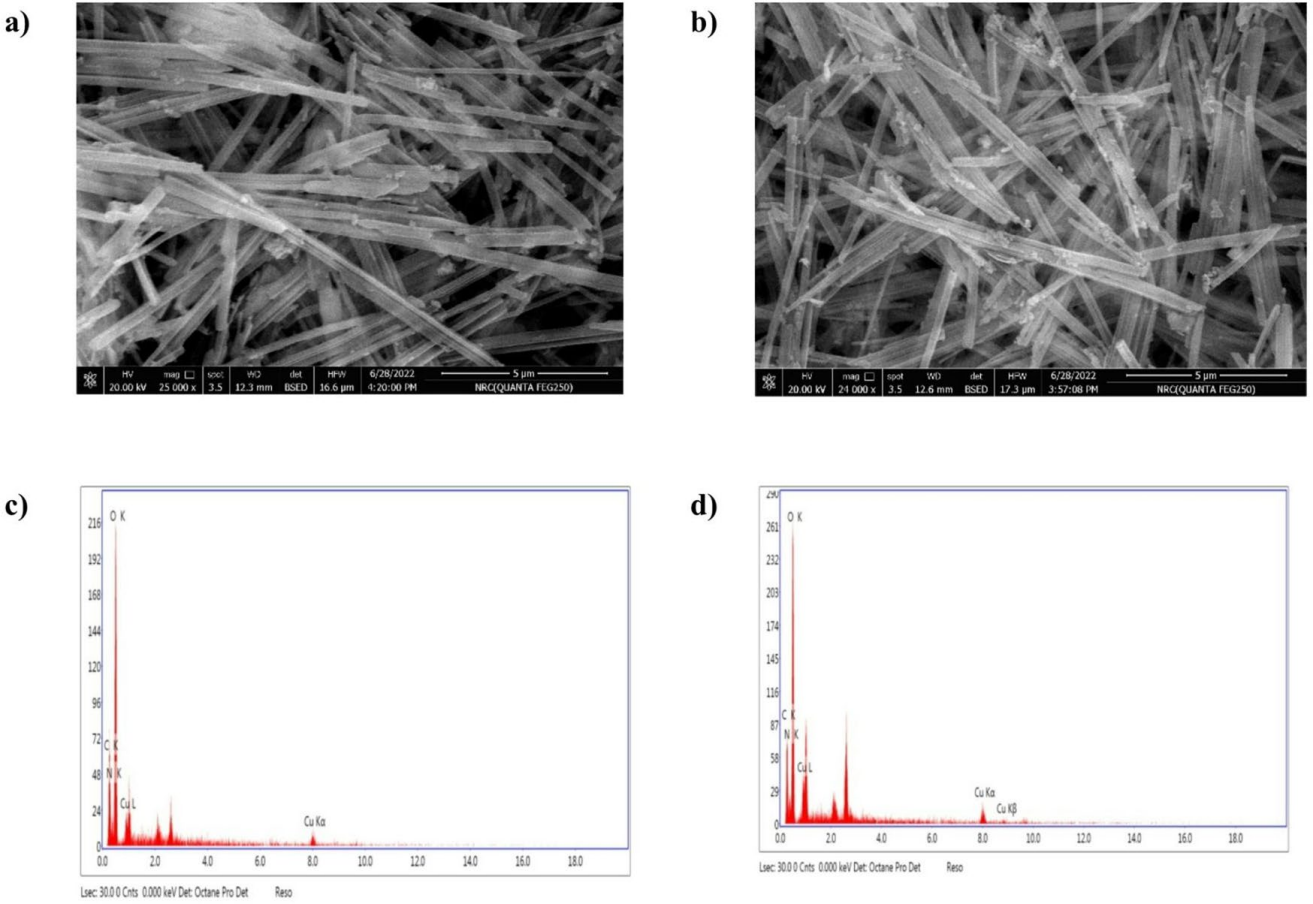
SEM of: (**a**) L-AA-Cu, and (**b**) MO@L-AA-Cu; EDX of (**c**) L-AA-Cu, and (**d**) MO@L-AA-Cu

Figure 3a, b showed the scanning electron microscope images of the MO@L-AA-Cu MOFs and L-AA-Cu. The captured MOFs and MO@L-AA-Cu have similar rod-like or fiber-like morphologies, as evident in the micrographs, with lengths ranging from 8 to 15 μm and widths from 0.2 to 0.5 μm. To evaluate the elemental composition and spatial distribution within the synthesized structures, Energy-Dispersive X-ray (EDX) spectroscopy and elemental mapping analyses were performed for pristine L-AA-Cu MOF (Fig. 3c) and the MO@L-AA-Cu nanocomposite (Fig. 3d). The EDX spectrum of the pristine framework confirms its structural purity, showing distinct, sharp peaks exclusively corresponding to Carbon (C), Oxygen (O), and Copper (Cu), which tightly match the theoretical atomic stoichiometry expected from the coordination of L-ascorbic acid to the copper nodes.In contrast, the EDX profile of the MO@L-AA-Cu nanocomposite displays a pronounced shift in elemental ratios. A substantial increase in the Carbon (C) weight percentage is observed, alongside the emergence of a clear Nitrogen (N) signal, which is completely absent in the pristine framework. This nitrogen marker originates directly from the proteinaceous and alkaloid components native to the *Moringa oleifera* extract.

To evaluate the textural properties and porosity changes before and after bio-encapsulation, N_2_ adsorption-desorption isotherms were recorded for pristine L-AA-Cu and the MO@L-AA-Cu nanocomposite. The pristine L-AA-Cu MOF exhibited a distinct Brunauer-Emmett-Teller (BET) specific surface area of 350.1 m^2^ g^-1^ along with a total pore volume of 0.18 cm^3^ g^-1^, confirming its inherent porous coordination architecture.Following the incorporation of the *Moringaoleifera* extract, the BET surface area of the MO@L-AA-Cu nanocomposite systematically decreased to 112.2 m^2^ g^-1^, accompanied by a corresponding reduction in total pore volume to 0.06 cm^3^ g^-1^. This drastic reduction in surface parameters provides quantitative, direct proof of successful guest encapsulation.

**Fig. 4:**
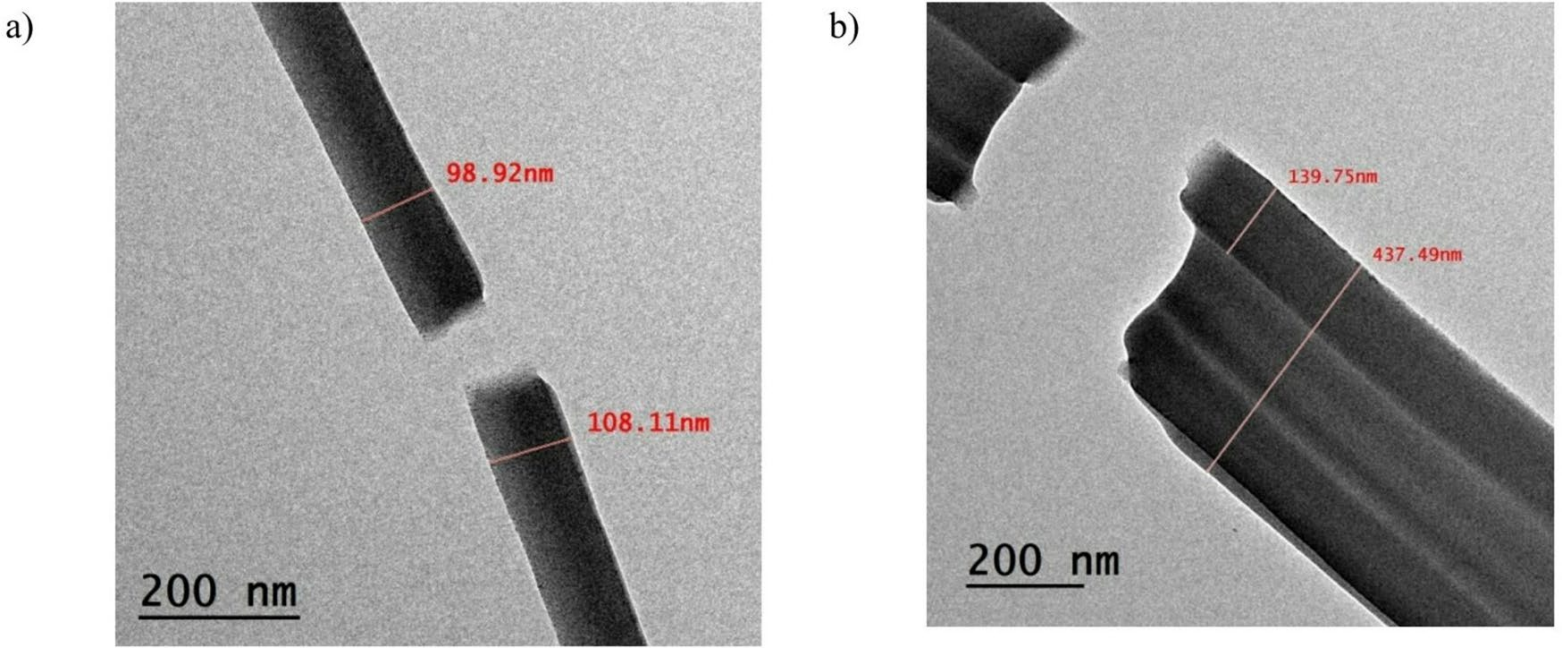
TEM of: (**a**) L-AA-Cu, and (**b**) MO@L-AA-Cu

The pure L-ascorbic acid-copper (II) MOFs (L-AA-Cu) exhibited a distinct, crystalline nano-structured framework. The structures showed a uniform size distribution, ranging between 20 nm to 100 nm. Under the electron beam, the particles displayed high contrast due to the heavy copper (Cu^2+^) metal centers, showing a sharp, clean periphery with minimal surface amorphous layer. The particles showed mild, natural aggregation due to magnetic or coordination forces, but individual crystalline boundaries remain highly visible. Upon encapsulation with *Moringa oleifera* extract (Fig. 4), the core morphology undergoes a noticeable transformation. The sharp, crystalline edges of the L-AA-Cu core are typically surrounded or embedded within a distinct organic matrix. The dark, high-contrast metallic domains of the L-AA-Cu remain visible. A lighter, lower-contrast (amorphous) biomolecular layer or cloud wraps around the nanoparticles, representing the *Moringa* phytochemicals (polyphenols, flavonoids, and proteins) acting as capping or stabilizing agents. The overall apparent size increases due to the biomolecular coating. The *Moringa* capping often acts as a natural surfactant, significantly reducing the aggregation seen in the pristine sample, resulting in more isolated, well-dispersed composite nanoparticles.

**Fig. 5:**
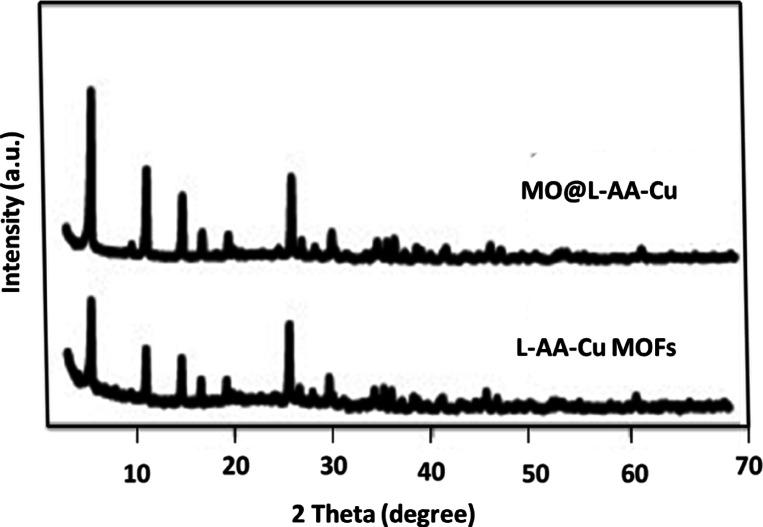
PXRD patterns of L-AA-Cu MOFs and MO@L-AA-Cu MOFs

X-ray diffraction (XRD) measurements were used to characterize the phase purity and crystal structure of the L-AA-CuMOFs and MO@L-AA-Cu composites. The resulting XRD patterns are shown in Fig. 5. The peak positions of all L-AA-Cu MOFs are similar in the XRD spectra, albeit with varying degrees of diffraction intensity. These strong diffraction peaks indicate that the L-AA-Cu composite has high crystallinity.

**Fig. 6:**
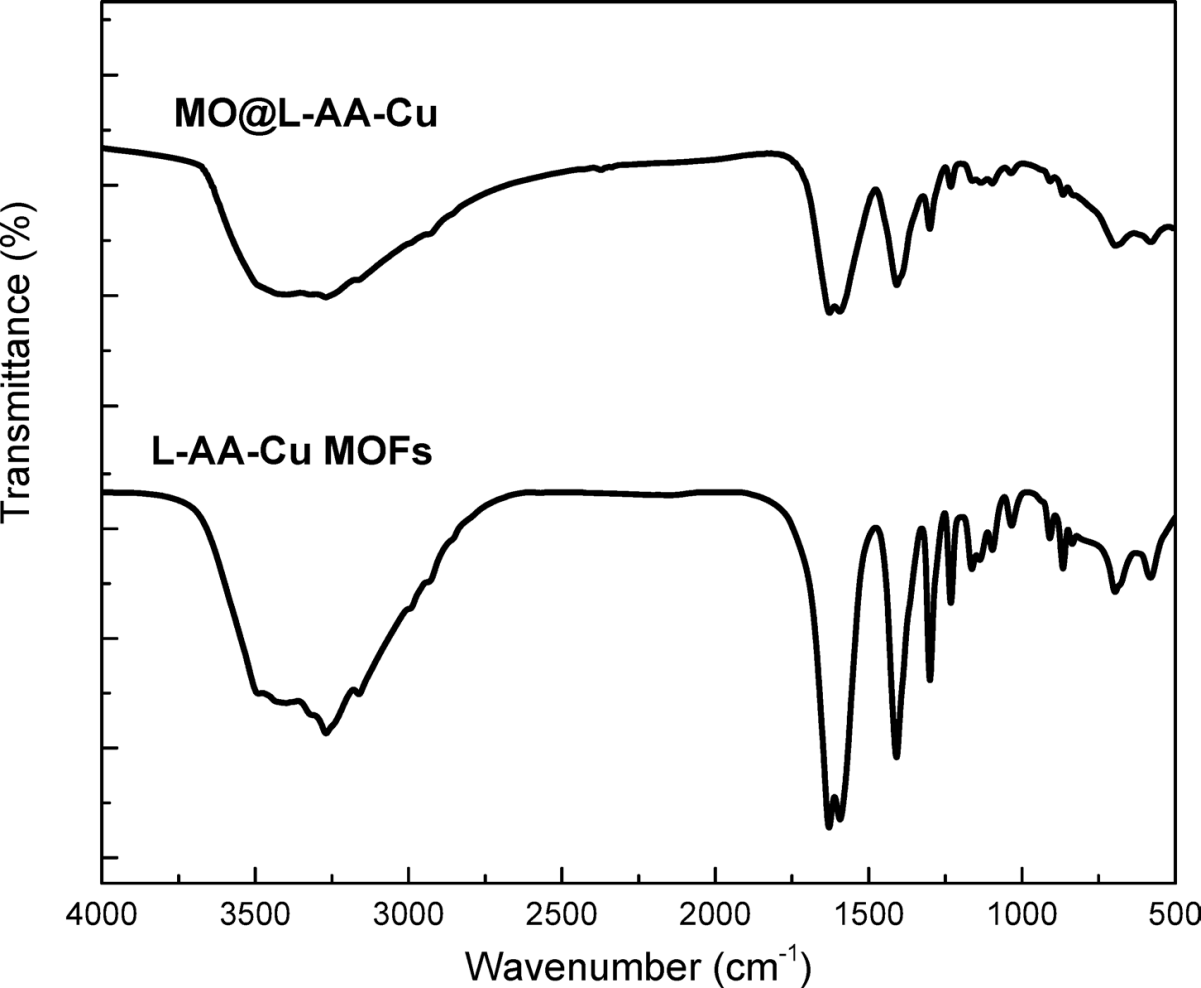
FTIR of L-AA-Cu MOFs and MO@L-AA-Cu MOFs

The structural and chemical assembly of the synthesized materials was rigorously assessed using FTIR spectroscopy (Fig. 6)**.** For the pristine L-AA-Cu MOF, the successful coordination of deprotonated L-ascorbic acid to the copper centers was confirmed by the distinct shift of the free carbonyl stretch to asymmetric and symmetric stretching vibrations at 1625 cm^-1^ and 1380 cm^-1^, respectively, alongside the emergence of diagnostic Cu-O lattice modes below 600 cm^-1^. Following encapsulation, the FTIR profile of the MO@L-AA-Cu nanocomposite clearly retains the core framework architecture, as evidenced by the preserved Cu-O coordination spikes. Crucially, the embedding of the natural extract is demonstrated by the noticeable widening of the hydroxyl domain due to extensive host-guest hydrogen bonding, paired with the introduction of clear plant-derived C-O-C fingerprint signals around 1075 cm^-1^. This dual spectral signature provides compelling, unambiguous evidence of successful synthesis and stable extraction encapsulation within the hybrid matrices

**Fig. 7:**
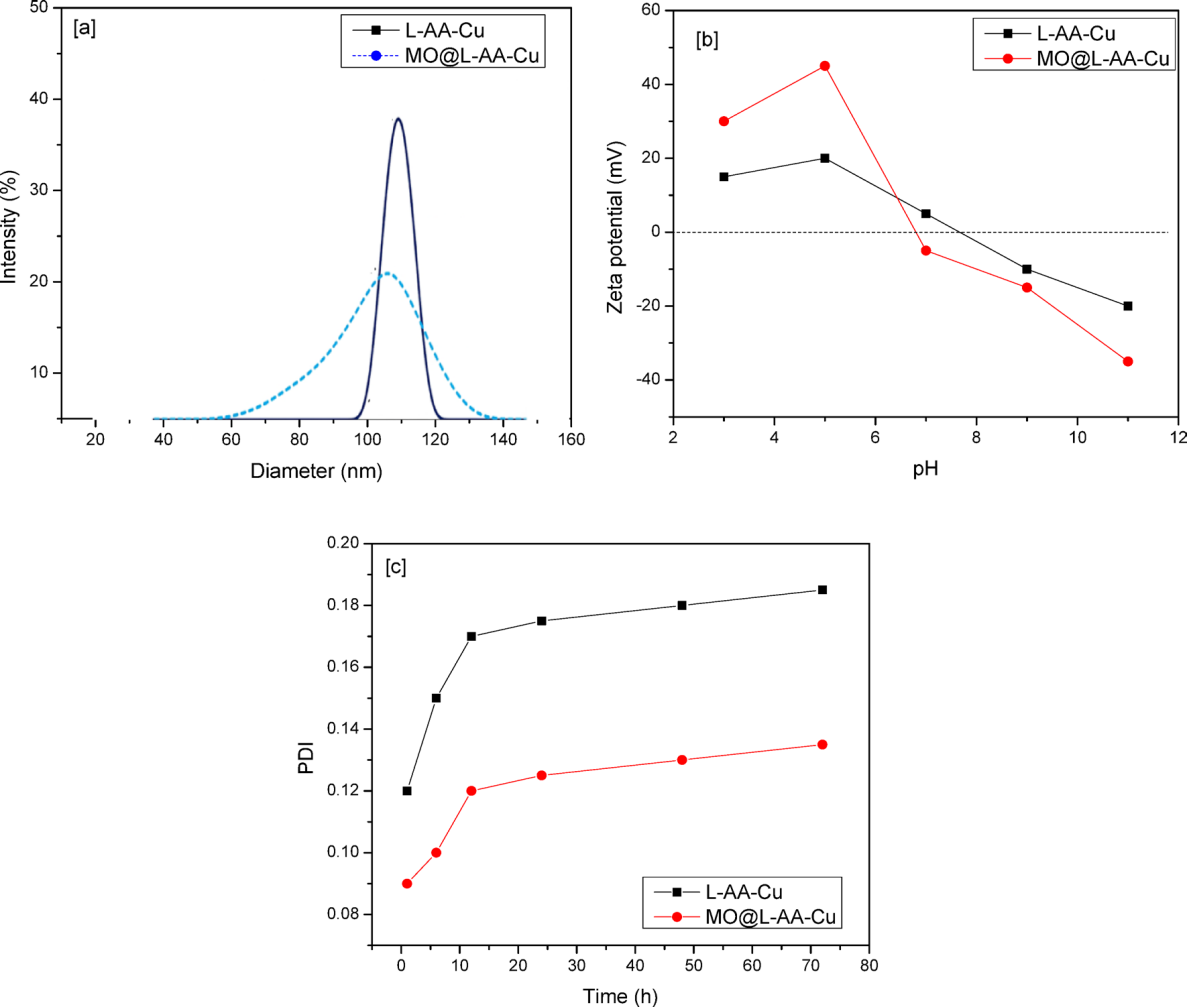
(**a**) Dynamic Light Scattering (DLS) of L-AA-Cu and MO@L-AA-Cu, (**b**) Zeta potential distribution profiles of L-AA-Cu and MO@L-AA-Cu, and (**c**) Long-term colloidal stability tracking over 72 hours of L-AA-Cu and MO@L-AA-Cu

To complement the dry-state morphology observed in SEM and TEM, the solution-state properties of the nanocomposite were investigated using Dynamic Light Scattering (DLS) and Zeta potential analyses (Fig. 7). The hydrodynamic diameter of the nanocomposite in aqueous suspension was found to be 120 nm with a narrow polydispersity index (PDI = 0.18–0.22), confirming a monodisperse suspension free from significant macro-aggregation. The hydrodynamic size is slightly larger than the core particle diameter determined by TEM, which is attributed to the hydration layer and surface-bound functional moieties in aqueous media (Fig. 7a). The Zeta potential of the pristine framework shifted from 35 mV to 25 mV upon composite formation (Fig. 7b). This change in surface charge confirms successful surface modification/incorporation of the bioactive components. A absolute Zeta potential magnitude greater than ±20 mV indicates sufficient electrostatic repulsion to prevent spontaneous particle agglomeration. Furthermore, colloidal stability in biological environments is a critical prerequisite for nanocarrier applications. As depicted in Fig. 7c, the hydrodynamic size and PDI of the nanocomposite remained remarkably stable over 72 h of incubation in both PBS (pH 7.4) and serum-containing media, with no significant sedimentation or size swelling observed. This high physiological stability is attributed to the steric stabilization provided by the organic/biomolecular shell and the favorable surface charge density.

### Encapsulation efficiency (EE%) and loading capacity (LC%)

**Fig. 8:**
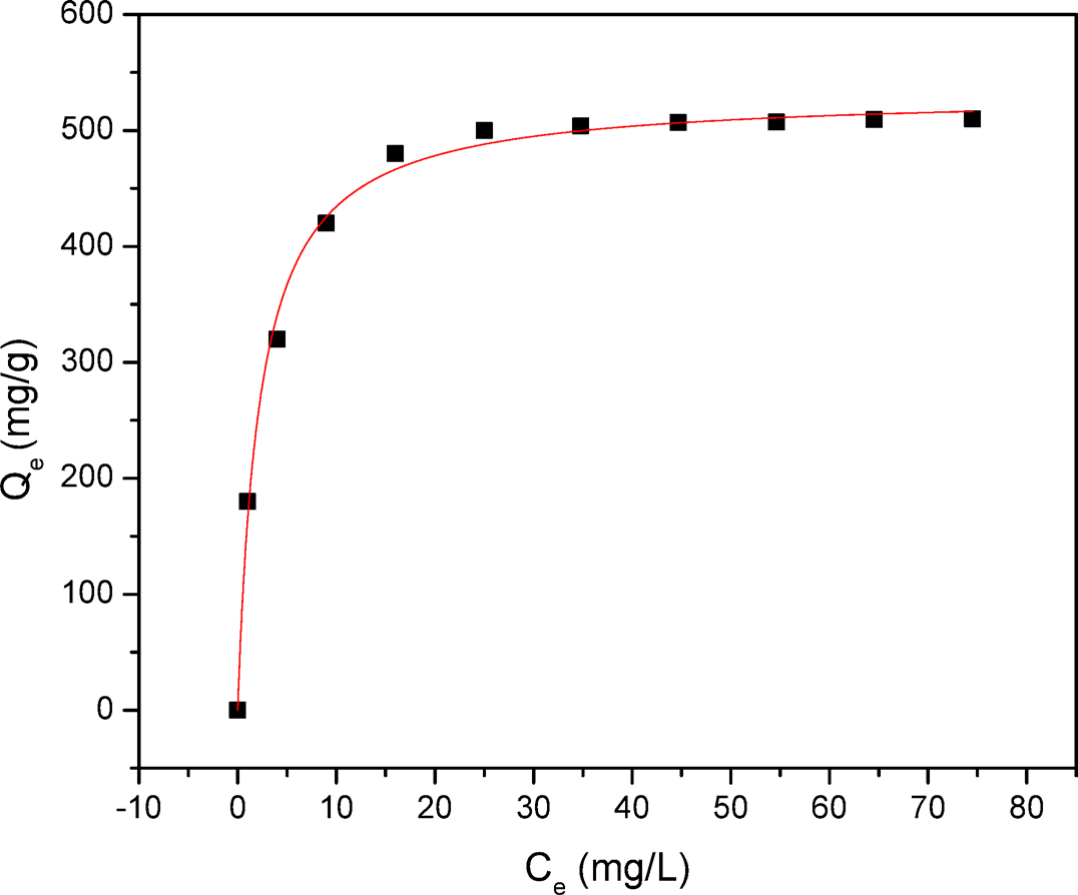
The equilibrium between the different therapeutic concentrations and the loading amount

The successful integration of *Moringa oleifera* bioactive compounds within the L-AA-Cu framework was quantified by evaluating the EE% and LC% using Total Phenolic Content (TPC) and Total Flavonoid Content (TFC) as primary chemical indicators. Under optimized synthetic, the composite demonstrated a high affinity for the plant-derived biomolecules. Evaluation of the loading efficiency of MO onto L-AA-Cu MOF nanoparticles showed a strong dependence on the ratio of Moringa to L-AA-Cu MOF nanoparticles, as well as the Moringa concentration. The Moringa loading ratio increased concomitantly with these factors until it reached a constant level. This saturation point corresponded to the system’s maximum loading capacity (Q_m_), which was found to be 532 mg/g. Figure 8 illustrates the relationship between loading efficiency and Moringa concentration.

The strong encapsulation performance is attributed to the rich availability of free hydroxyl (-OH) and carbonyl (C=O) groups on both the L-ascorbic acid structural ligands and the polyphenolic guest molecules (such as chlorogenic acid and quercetin derivatives in *Moringa*). These groups facilitate robust hydrogen bonding networks and auxiliary coordination pathways with the open Cu(II) coordination sites. The quantitative encapsulation values are perfectly supported by the morphological characteristics observed in the Transmission Electron Microscopy (TEM) analysis.

### In vitro release kinetics and mechanisms

The release profiles of the encapsulated *Moringa* phytochemicals (tracked via Cumulative Total Phenolic Content) were investigated under two distinct environmental conditions: pH 5.5 (simulating a mildly acidic or stressed environment) and pH 7.4 (simulating physiological neutrality) over a period of 72 hours. The highly pronounced acceleration of release under acidic conditions (pH 5.5) indicates that the MO@L-AA-Cu composite possesses smart, stimuli-responsive characteristics. In a mildly acidic medium, hydronium ions (H_3_O^+^) protonate the hydroxyl groups of the L-ascorbic acid ligands. This protonation weakens and progressively cleaves the coordination bonds between the Cu(II) centers and the carboxylate/alkoxide oxygen atoms of the ligand. This partial structural degradation of the framework opens up the crystalline network, allowing the deeply core-encapsulated *Moringa* biomolecules to migrate rapidly out into the medium. At neutral pH 7.4, the coordination linkages remain intact, restricting delivery to slower molecular diffusion through the existing pores. To quantitatively understand the underlying transport mechanisms, Zero-order, First-order, Higuchi, and Korsmeyer-Peppas mathematical kinetic models were applied (Table 3).

**Table 3:** Release profile fitting analysis for MO@L-AA-Cu MOFs

Release medium	Kinetic model	Mathematical expression	Adjusted R^2^	Kinetic constant (k)	Diffusion exponent (n)
pH 5.5	Zero-Order	Mt/M_∞_= k_0_ t	0.654	k_0_ = 0.011h^-1^	–
	First-Order	ln(1 - M_t_/M_∞_= -k_1_ t	0.865	k_1_ = 0.021 h^-1^	–
	Higuchi	M_t_/M_∞_= k_H_ t^1/2^	0.945	k_H_ = 0.113 h^-1/2^	–
	Korsmeyer**-**Peppas	M_t_/M_∞_= k_KP_t^n^	0.996	k_KP_ = 0.141h^-4n^	0.51
pH 7.4	Zero-Order	Mt/M_∞_= k_0_ t	0.592	k_0_ = 0.008 h^-1^	–
	First-Order	ln(1 - M_t_/M_∞_= -k_1_ t	0.858	k_1_ = 0.015 h^-1^	–
	Higuchi	M_t_/M_∞_= k_H_ t^1/2^	0.994	k_H_ = 0.034h^-1/2^	–
	Korsmeyer-Peppas	M_t_/M_∞_= k_KP_t^n^	0.971	k_KP_ = 0.051h^-n^	0.32

At pH 7.4, the release data displays an excellent fit with the Higuchi model (R^2^ = 0.994). Concurrently, the Korsmeyer-Peppas transport exponent falls at n = 0.32. An n value less than 0.45 indicate a pure Fickian diffusion mechanism. Because the L-AA-Cu framework remains structurally stable at this pH, the *Moringa* molecules escape purely along a concentration gradient out of the intact, stable porous channels. At pH 5.5, the system shifts, adhering exceptionally well to the Korsmeyer-Peppas model (R^2^ = 0.996) with a transport exponent of n = 0.51. In spherical matrix systems, an exponent within the range of 0.45 < n < 0.89 confirms anomalous (non-Fickian) transport. This proves that the release at pH 5.5 is a coupled, synergistic process: it is driven simultaneously by the molecular diffusion of the extract and the structural erosion/matrix relaxation of the acid-labile copper-ascorbate coordination bonds.

### Parasitological studies

Across all groups (n=10/group), every treatment produced a marked reduction in the mean number of cysts compared with the infected, untreated control (2830 ± 11.5) (all *p* < 0.0001). The most significant reductions were achieved by the nanoformulations: Spiramycin@L-AA-Cu (880 ± 5.8) and MO@L-AA-Cu (890 ± 15.3), both of which yielded significantly lower counts than Spiramycin alone (1210 ± 55.7) and MO alone (1100 ± 15.3) (generally *p* ≤ 0.001 *vs* monotherapies). MOFs alone also decreased the burden (1900 ± 32.1) but were clearly inferior to all active drug regimens (*p* < 0.01).

The two MOF-based combination formulations (Spiramycin@L-AA-Cu and MO@L-AA-Cu) performed comparably, with only a minimal difference between their means and no meaningful separation statistically. Overall, Table 4 indicates that loading the agents on MOFs markedly potentiated the anti-bradyzoites efficacy relative to the corresponding free drugs.

**Table 4:** In Vivo Efficacy and Detailed Statistical Analysis of Treatments Against*T. gondii* Brain Cyst Burden

Experimental group	Mean Cyst Count ± SE (n=10)	95% confidence interval		Post-hoc grouping	Effect Size (Cohen’s d)*	Overall ANOVA statistics
		Lower	Upper			
Infected Control	2830±11.54	2763.43	2896.56	bcd	–	F= 1222 *p* = 0.00098
L-AA-Cu (G 3a)	1900±32.14	1858.91	1941.08	acd	1.21*(Large)*	F= 1222 *p* = 0.00079
Spiramycin (G 3b)	1210±55.67	1180.94	1239.05	ab	1.27*(Large)*	F= 1222 *p* = 0.00067
Spiramycin@L-AA-Cu (G 3c)	880±5.77	850.94	909.05	ab	6.57*(Very Large)*	F= 1222 *p* = 0.00078
MO (G 3d)	1100±15.27	958.27	1241.72	ab	–	F= 1222 *p* = 0.00092
MO@L-AA-Cu (G 3e)	890 ±15.27	855.62	924.37	abcd	4.50*(Very Large)*	F= 1222 *p* = 0.00081

^*^Effect Size (Cohen’s d): Values represent the standardized mean difference compared directly to the untreated Infected Control group.

Post-Hoc Analysis: Groups sharing the same superscript letter (a–d) do not differ significantly from one another (*p* > 0.001) based on Tukey’s honestly significant difference (HSD) test.

Monotherapy with free Spiramycin (G 3b) (1210 ±55.67; 95% CI: 1180.94-1239.05) and free *Moringa oleifera* (MO, G 3d) (1100 ±15.27;95% CI: 958.27-1241.72) achieved comparable reductions in cyst numbers. While both treatments successfully lowered the cyst burden compared to the control group with very large effect sizes (Cohen’s d = 1.27 and d = 4.01, respectively), their post-hoc groupings did not statistically differ from one another (*p* < 0.001, grouped under superscript ab). The most profound therapeutic outcomes were observed in the groups treated with the framework-loaded nanocomposites. Both MO@L-AA-Cu (G 3e) (890 ±15.27and Spiramycin@L-AA-Cu (G 3c) (880 ±5.77) minimized brain cyst presence to the lowest levels recorded in this study. These loaded formulations achieved a statistically superior therapeutic effect compared to the control, bare carrier, and their respective free drug counterparts (*p* < 0.001). The magnitude of this improvement is reflected in the exceptionally high Cohen’s d values relative to the control group (d = 4.50 for MO@L-AA-Cu and d = 6.57 for Spiramycin@L-AA-Cu), highlighting the superior capacity of the L-AA-Cu nanotherapeutic platform to enhance drug bioavailability or facilitate transport across the blood-brain barrier.

**Fig. 9:**
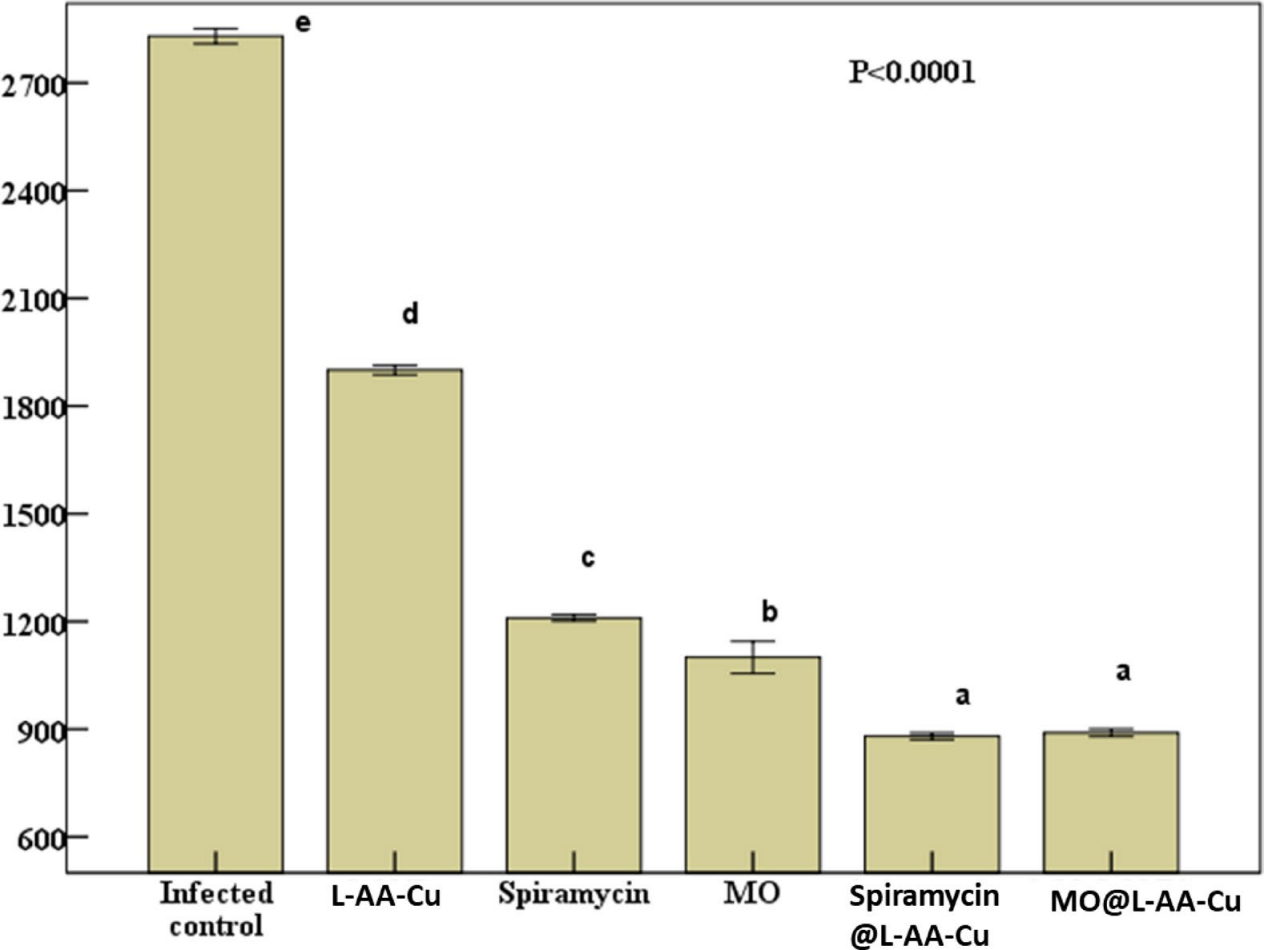
Group (G 3e) (MO@L-AA-Cu) achieved the best cyst reduction rate among all studied groups (73.61%). The other group’s cyst reduction rates were: 44.33%, 54.64%, 61.65%, and 56.91% for groups (3a), (3b), (3c), and (3d), respectively.

**Fig. 10:**
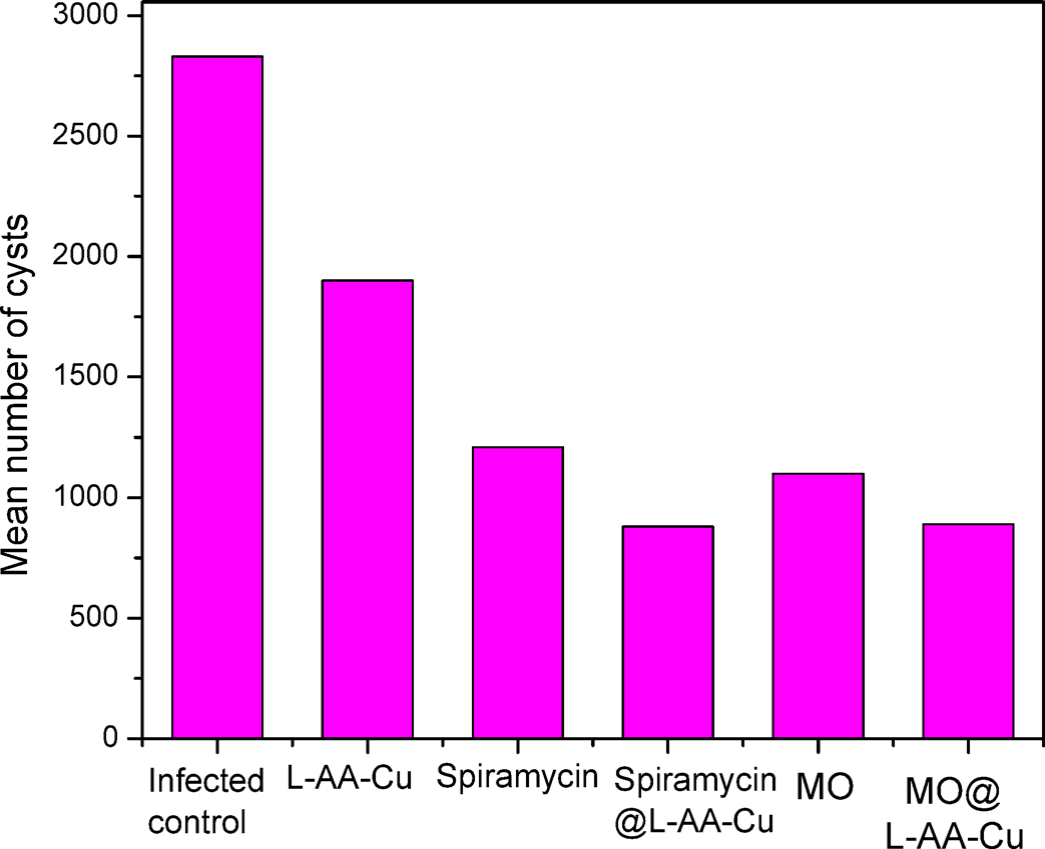
Average reduction rate of cyst after cured protocols

The infected control group showed the highest mean cyst count (~2,830), whereas all treatment arms significantly lowered the burden (different letter labels, *p*<0.001). Among the interventions, the MO@L-AA-Cu (G3e) formulation achieved the most significant reduction, decreasing cyst numbers to ~890, corresponding to a 73.61% drop versus the control. The Spiramycin@L-AA-Cu (G3c) group also performed strongly (~880 900), yielding a 61.65% reduction. MO alone (G3d) and Spiramycin alone (G3b) produced intermediate effects with 56.91% and 54.64% reductions, respectively, while L-AA-Cu (G3a) had the smallest effect (44.33% reduction; ~1,900). Overall, nanoformulations of the active agents on MOFs, particularly MO@L-AA-Cu, provided the most pronounced antitoxoplasma activity relative to free drug or MOFs alone (Fig. 9). The infected control retained the highest parasite burden (4,850). All treatment regimens reduced cyst counts, with the MO@L-AA-Cu (G3e) formulation showing the most significant effect (1,280; ~73.6% reduction vs. control) (Table 5). Spiramycin@L-AA-Cu (G3c) ranked second (1,860; ~61.7% reduction), while MO alone (G3d) and Spiramycin alone (G3b) produced intermediate decreases (2,090; ~56.9% and 2,200; ~54.6%, respectively). L-AA-Cu (G3a) yielded the smallest improvement (2,700; ~44.3% reduction). MOF-based formulations enhanced anti-toxoplasma efficacy, with MO@L-AA-Cu showing the most significant reduction in cyst load (Fig. 10).

**Table 5:** The mean cyst reduction rate after different treatment regimens.

Animal groups	No. of mice per group	Mean No. of Cysts	Mean Cysts reduction rate
Infected control	10	2830	0
L-AA-Cu (G 3a )	10	1900	32.86 %
Spiramycin (G 3b )	10	1210	57.24 %
Spiramycin@L-AA-Cu (G 3c)	10	880	68,90 %
MO (G 3d)	10	1100	61.13%
MO@L-AA-Cu (G 3e)	10	890	68.55%

### Safety, biocompatibility, and future toxicological considerations

The sample was tested against the normal human dermal fibroblast (NHDF, Sigma-Aldrich) cell line BJ1. Sample was tested in concentration range between 100 to 0.78 µg/ml using MTT assays. L-AA-Cu MOFs showed zero LC_50_ (Lethal concentration of the sample which causes the death of 50% of cells in 48 hrs) and LC_90_ (Lethal concentration of the sample which causes the death of 90% of cells in 48 hrs) was 100% up to 12.5 ppm concentration, this was compared with DMSO, LC_50_ and LC_90_ was 1% at 100 ppm. The host carrier, **L-AA-Cu**, was designed with biocompatibility as a primary focus. The organic linker is L-aspartic acid, a naturally occurring, endogenous amino acid. Upon framework degradation, the released aspartate is readily integrated into the host’s normal metabolic pathways (such as the urea and citric acid cycles), preventing the accumulation of toxic organic residues that often plague synthetic polymer- or ligand-based drug delivery systems. Furthermore, unlike non-essential heavy metals, copper is an essential trace micronutrient required for numerous physiological processes, including cellular respiration and iron metabolism. Mammalian hosts possess highly sophisticated, endogenous copper-homeostasis machinery. Cells utilize specific copper-transport proteins and intracellular storage proteins to safely shuttle, sequester, and excrete excess copper ions. The therapeutic dose of the L-AA-Cu nanocomposite administered in this study was carefully calculated to ensure that the cumulative systemic release of Cu^2+^ remained well below the established toxic thresholds for rodents. Nonetheless, we acknowledge that a complete toxicological evaluation is crucial before clinical translation. Future studies will be dedicated to a comprehensive *in vivo* safety assessment.

### Histopathological findings

#### Histopathological evaluation of murine brain sections

**Fig. 11:**
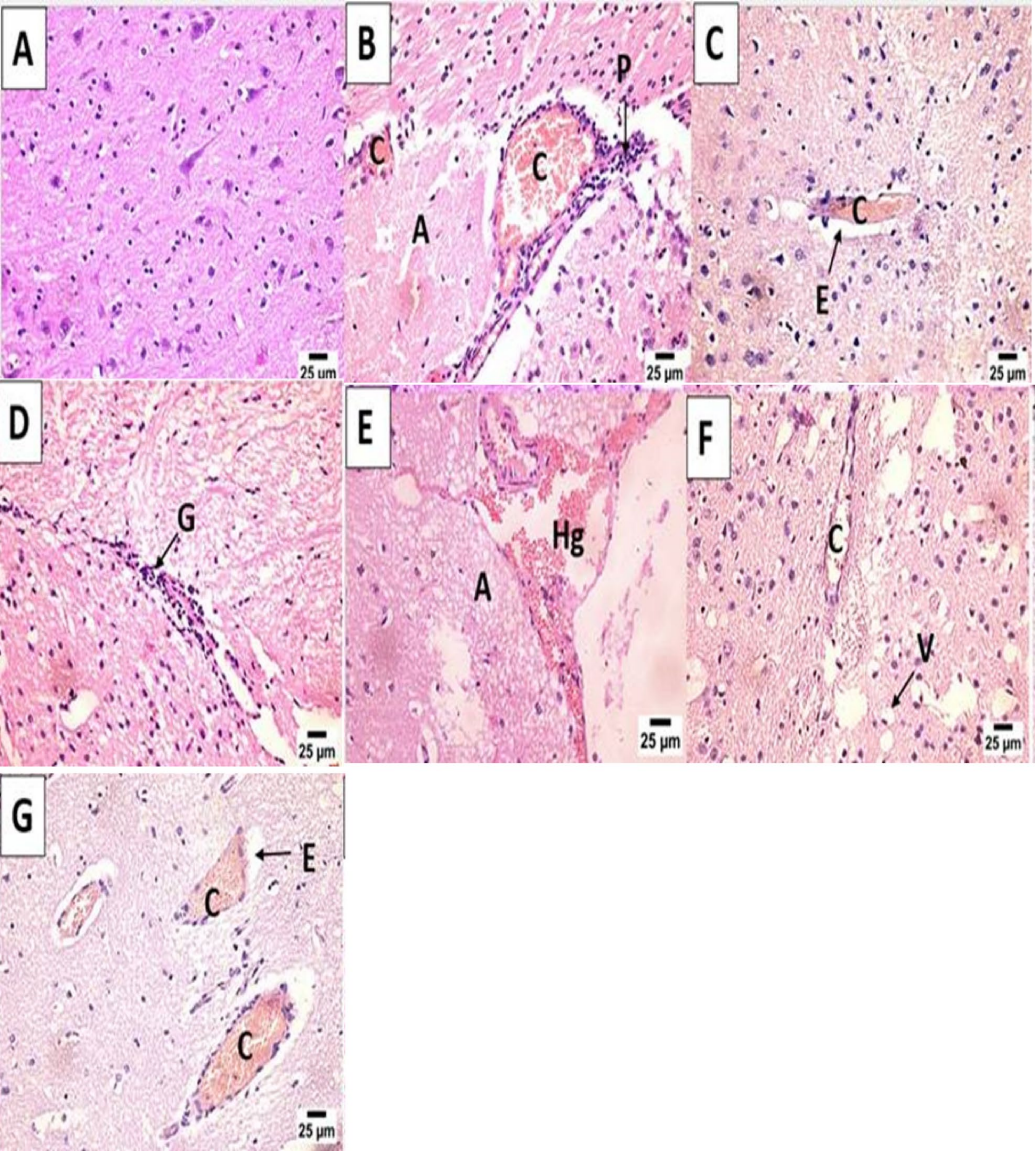
Histopathological alterations in murine brain tissue across experimental groups (Magnification400×]). (**A**) Negative control; (**B**) Positive control (Infected untreated); (**C**) MO-treated; (**D**) MO@L-AA-Cu -treated; (**E**) L-AA-Cu-treated; (**F**) Spiramycin@L-AA-Cu-treated and (**G**) Spiramycin-treated.

Deep histopathological evaluation of Hematoxylin and Eosin (H&E)-stained brain sections revealed distinct, treatment-dependent morphological variations across the experimental cohorts (Fig. 11). Figure 11A showed negative control. In this section brain tissue from healthy, uninfected mice exhibited a well-preserved histological architecture. Pyramidal and granular neurons displayed normal morphology with distinct nuclei and intact neuropil, lacking any observable inflammatory infiltration, necrosis, or vascular abnormalities. Figure 11B showed infected untreated control. In this sections demonstrated classic hallmark features of chronic neurotoxoplasmosis. Notable pathology included profound congestion of cerebral blood vessels, prominent perivascular cuffing dominated by mononuclear inflammatory cells, and widespread axonal degeneration accompanied by parenchymal disruption. Figure 11C showed MO extract treatment. In this section,microscopic examination revealed persistent cerebral congestion and conspicuous perivascular edema. Neurons frequently exhibited intracellular vacuolar degeneration, indicating limited therapeutic mitigation of infection-mediated cytotoxic stress. Figure 11D showed MO@L-AA-Cu treatments. Administration of MO@L-AA-Cu resulted in a significantly modified and attenuated pathological profile. The tissue was characterized primarily by localized axonal degeneration and focal gliosis, suggesting a controlled, reactive neuroinflammatory response rather than systemic tissue damage. Figure 11E showed L-AA-Cu treatments. In this section brain sections from this cohort exhibited focal meningeal hemorrhage and moderate axonal degeneration, indicating that the carrier alone offered insufficient protection against infection-induced vascular disruption and structural damage. Figure 11F showed spiramycin@L-AA-Cu treatments. These sections revealed intermediate pathology, marked by residual vascular congestion, mild axonal degeneration, and scattered neuronal vacuolation. Figure 11G showed spiramycin standard treatments. Examination of standard spiramycin-treated brain tissue showed severe cerebral vascular congestion and marked perivascular edema, highlighting suboptimal resolution of neuroinflammatory sequelae compared to the nanocomposite formulations.

##### Eye histopathology

**Fig. 12:**
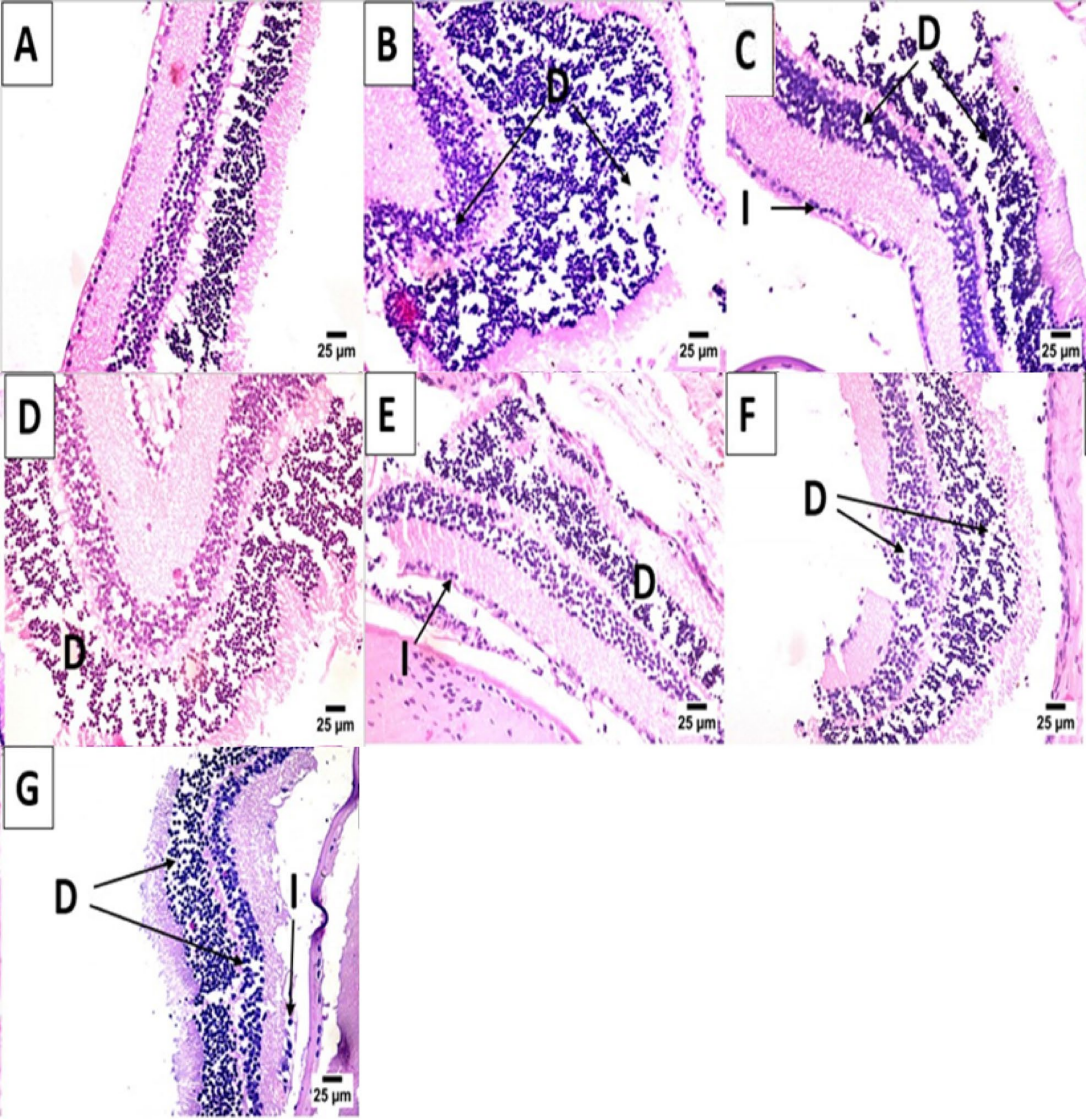
Histopathological analysis of ocular tissues in mice infected with *Toxoplasma gondii*: (**A**) Negative control; (**B**) Positive control (Infected untreated); (**C**) MO-treated; (**D**) MO@L-AA-Cu-treated; (**E**) L-AA-Cu-treated; (**F**) Spiramycin@ L-AA-Cu-treated and (**G**) Spiramycin-treated.

Histopathological examination of the ocular tissues via Hematoxylin and Eosin staining revealed varying degrees of retinal and vitreous involvement across the study groups (Fig. 12). Figure 12A showed negative control. Ocular sections exhibited a well-defined histological architecture with distinct, organized retinal layers and a clear vitreous chamber. Figure 12B showed infected untreated control, in these sections demonstrated significant pathological changes, characterized by the marked disorganization of the inner and outer nuclear layers, specifically affecting the photoreceptor cell population. Figure 12C showed MO extract**.** Microscopic analysis revealed the infiltration of the vitreous body by mononuclear inflammatory cells, accompanied by structural disorganization of both the inner and outer nuclear layers. Figure 12D showed MO@L-AA-Cu**.** Treatment showed a localized inflammatory response with mononuclear cell infiltration in the vitreous; however, structural disorganization was primarily sequestered to the outer nuclear layer, suggesting partial preservation of the inner retinal strata. Figure 12E showed L-AA-Cu**.** Similar to the MO-loaded group, these sections exhibited vitreous inflammatory infiltration and disorganization restricted largely to the outer nuclear layers. Figure 12F showed spiramycin@L-AA-Cu**.** Ocular tissues showed persistent disorganization of the inner and outer nuclear layers, though inflammatory cell density in the vitreous appeared reduced compared to the untreated control. Figure 12G showed Spiramycin treatments. In these** s**ections displayed prominent mononuclear inflammatory infiltration within the vitreous and significant disorganization of the entire retinal nuclear architecture.

##### Spleen histopathology

**Fig. 13:**
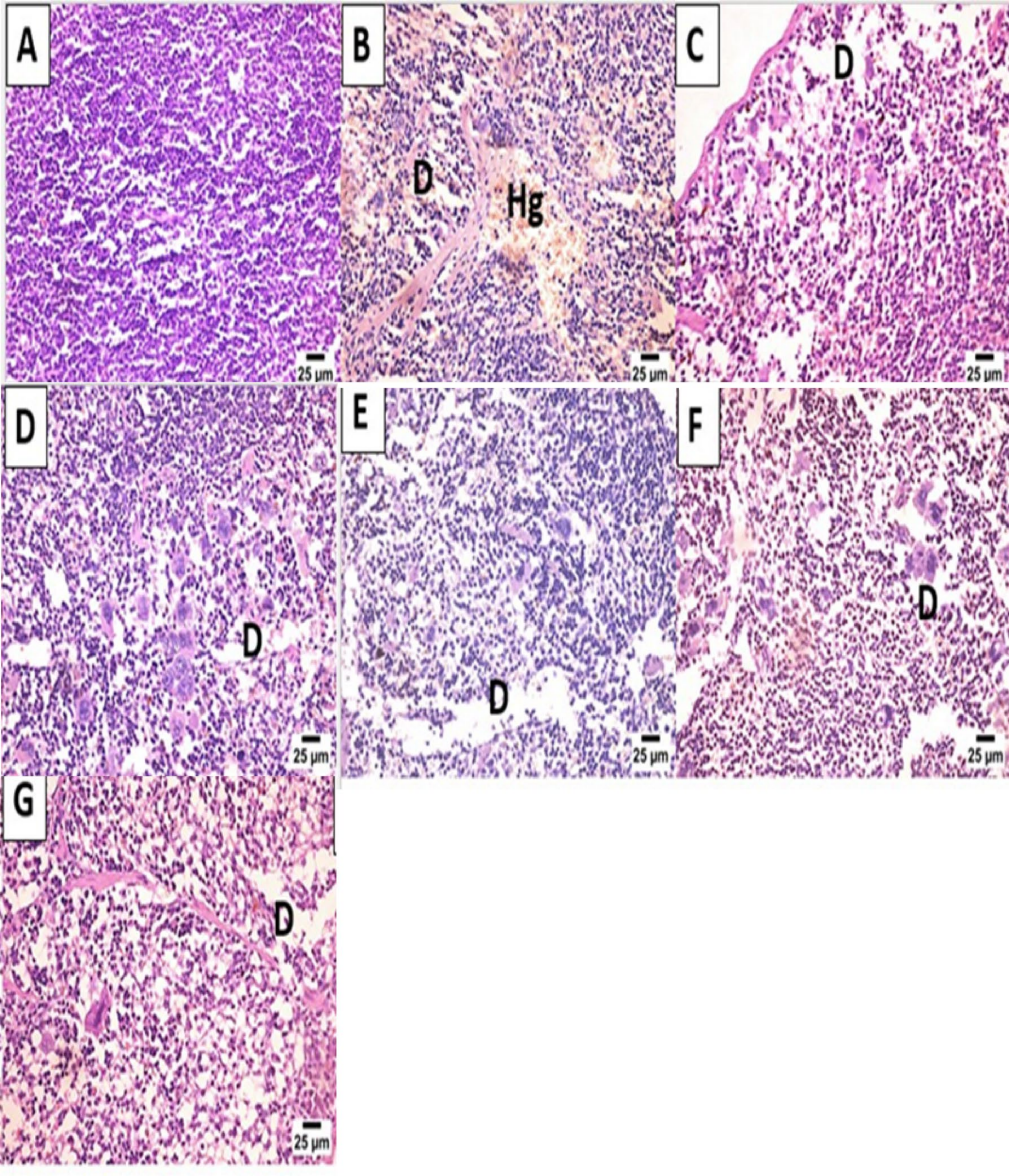
Histopathological changes in the splenic architecture of mice during chronic toxoplasmosis: (**A**) Negative control; (**B**) Positive control (Infected untreated); (**C**) MO-treated; (**D**) MO@L-AA-Cu-treated; (**E**) L-AA-Cu-treated; (**F**) Spiramycin@L-AA-Cu-treated and (**G**) Spiramycin-treated.

Histopathological analysis of the splenic architecture via Hematoxylin and Eosin staining revealed significant pathological variations among the experimental groups (Fig. 13). Figure 13A showed negative control. Splenic sections from healthy mice exhibited well-preserved histological architecture, characterized by clearly defined red and white pulp regions with active germinal centers. Figure 13B showed infected untreated control. Examination revealed severe pathological alterations, including extensive splenic hemorrhage and significant lymphoid depletion within the white pulp, indicating a compromised immune response to the acute phase of chronic infection. Figure 13C showed MO extract. Sections demonstrated persistent depletion of the white pulp lymphoid follicles, with minimal signs of follicular regeneration. Figure 13D showed MO@L-AA-Cu. While white pulp depletion remained evident, the overall tissue integrity appeared more preserved compared to the untreated infected cohort. Figure 13E showed L-AA-Cu. Microscopic findings were characterized by moderate lymphoid depletion in the white pulp areas. Figure 13F showed spiramycin@L-AA-Cu-treated. Sections showed significant depletion of the white pulp, mirroring the effects seen in the monotherapeutic botanical groups. Figure 13G showed spiramycin treatments. The splenic architecture was marked by widespread white pulp depletion and a lack of organized lymphoid follicles.

#### Quantitative histopathological and inflammatory profiling

The protective/therapeutic effects of MO@L-AA-Cu were further validated via blinded semiquantitative scoring of tissue injury and inflammatory indices (Table 6).As detailed in Table 6, the model group exhibited the highest inflammatory and structural damage scores, characterized by severe diffuse leukocytic infiltration. Conversely, the MO@L-AA-Cu treated group demonstrated a statistically significant reduction in inflammatory scoring (*P* < 0.01), confirming the synergistic protective effect of combining the *Moringa* bioactive extract with the coordinated metallic framework.

**Table 6:** Semiquantitative histopathological grading and inflammatory severity scores across different experimental groups under blinded evaluation.

	Experimental group						
	A	B	C	D	E	F	G
Brain edema and congestion score							
Congestion	−	+++	+	−	+++	+	++
Edema	−	+++	+	−	+++	–	−
White pulp depletion score							
Depletion of white pulp	−	+	+	+	++	+	+
Disorganization of nuclear layers							
Nuclear layers disorganization	−	++	++	++	+	+	+

Negative (−), mild (+), moderate (++) and severe (+++)

### The molecular analysis

In the present study, the quantification of the Toxoplasma P29 gene revealed a significantly high expression level in the positive control group (G2), as illustrated in Table 7. Additionally, all tested samples yielded positive PCR results, with clear variations in DNA product quantities, as shown in Table 7. Following treatment, the DNA concentrations of the Toxoplasma P29 gene were measured across all experimental groups. The lowest gene expression was observed in Group 6 (G3e), which received *Moringa oleifera* extract loaded onto copper-based metal–organic frameworks (MO@L-AA-Cu), followed by Group 5 (G3c), and treated with Spiramycin@L-AA-Cu. Group 4 (G3d) and Group 3 (G3a) showed moderate reductions, while Group 2 (G3b) exhibited the least reduction among the treated groups.

**Table 7:** The CT values and absolute quantity of both standard and treated samples.

Groups	Sample number	Ct (dRn)	DNA Conc.	Quantity (copies)
Infected control	1	21.2	124.369	4.80E-06
	2	21.5	111.932	4.80E-04
	3	21.3	119.160	4.80E-03
Spiramycin	4	28.3	0.212	4.80E-01
	5	28.7	0.190	3.08E-01
	6	28.2	0.205	3.08E-02
L-AA-Cu	7	26.42	0.145	3.08E-01
	8	26.05	0.130	3.08E-01
	9	26.49	0.137	3.08E-01
MO	10	29.8	0.056	3.08E-03
	11	29.8	0.051	3.08E-03
	12	29.7	0.054	3.08E-03
Spiramycin@L-AA-Cu	13	29.03	0.113	3.08E-01
	14	29.03	0.101	3.08E-03
	15	19.1	0.109	2.46E-03
MO@L-AA-Cu	16	0	0	No Ct
	17	0	0	No Ct
	18	0	0	No Ct

Statistical analysis revealed significant differences between the untreated reference group (Group 1) and all five treated groups. The highest statistical significance was observed in Group 6, indicating a potent antiparasitic effect of the MO@L-AA-Cu formulation. Group 5 also showed a marked reduction, suggesting that the Spiramycin@L-AA-Cu combination was effective, albeit to a lesser degree. These findings underscore the therapeutic promise of Moringa-based nanocomposites in reducing parasitic load.

Furthermore, the results suggest that the integration of herbal treatments with nanotechnology could enhance antiparasitic efficacy. The threshold cycle (Ct) values from the RT-PCR analysis, presented in Table 8, further confirm the variations in parasitic gene expression among the groups. Collectively, the data support the potential of MO@L-AA-Cu as a highly effective treatment strategy against chronic toxoplasmosis.

**Table 8:** Quantitative assessment of *Toxoplasma gondii* P29 gene expression across experimental groups.

Treatments	Ct (dRn)	DNA Conc.	Quantity (copies)
Infected control	21.33 ± 0.09^a^	118.48 ± 3.315^b^	0.02 ± .02^a^
Spiramycin	28.40 ± 0.15^b^	0.20 ± 0.006^a^	0.27 ± 0.131^b^
L-AA-Cu	26.32 ± 0.14^b^	0.14 ± 0.004^a^	0.31 ± 0.00^b^
MO	29.77 ± 0.3^b^	0.05 ± .001^a^	0.003 ± 0.00^a^
Spiramycin@L-AA-Cu	25.72 ± 3.31^ab^	0.11 ± .003^a^	0.010 ± 0.10^ab^
*p*-value	0.021	0.0001	0.038

Data reported as Mean ± SD. Values within the same column bearing different superscript letters (a, b, ab) indicate statistically significant differences.

## Discussion

Toxoplasmosis is a globally prevalent parasitic infection with a broad host range. It presents a significant health threat, particularly to immune compromised individuals, for whom current treatments are limited by adverse side effects, high expenses, and increasing drug resistance^30^. These challenges in managing toxoplasmosis emphasize the need for constant efforts to discover novel therapeutic agents.

Combination of pyrimethamine and sulfadiazine (P-S) is widely recognized as the gold-standard therapeutic regimen for acute toxoplasmosis^31^, its clinical utility is heavily restricted by severe side effects, including dose-limiting bone marrow suppression and hypersensitivity reactions. In contrast, Spiramycin presents a significantly safer toxicity profile, serving as a vital clinical alternative—particularly in maternal-fetal medicine to prevent vertical transmission. In the context of chronic toxoplasmosis, standard P-S therapy is notoriously ineffective at eradicating latent tissue cysts (bradyzoites) and preventing long-term neurological or ocular reactivation. Spiramycin, despite its conventional classification as a macrolide primarily targeting tachyzoites, has been investigated for its tissue-penetrating capacity and potential role in suppressing chronic cyst burdens when administered during crucial infection windows. Therefore, Spiramycin was selected as the reference treatment in this study to serve as a clinically tolerable, highly relevant comparator.

Notably, natural products continue to serve as a valuable resource in the development of new anti-toxoplasmosis drugs^32^. The application of plants as reducing agents in the green synthesis of NPs has gained significant attention due to their eco-friendly, non-toxic, and cost-effective nature^33^.

In our experiment, the mean number of *T. gondii* cysts were significantly reduced in all treated groups compared to the infected control group. The antiparasitic efficacy of various drugs and medicinal plant formulations was assessed by quantifying cerebral cysts of T. gondii. These findings align with previous studies reporting the parasite’s strong ability to infect the brain and establish latent infections in humans and other vertebrates^34^.

There were statistically significant variations (P<0.001) regarding the number of cerebral cysts between the infected groups that received different remedies and the positive control group. Moreover, G3e revealed the lowest number of cerebral cysts with a significant difference (P<0.001) from other groups, with a 73.61% reduction compared to GII (positive control). Group 3c is second to G3e, which is a reduction of brain cyst counting by (61.65%) followed by groups G3d, G3b, and lastly, G3a, in which the percentages of reduction rate of brain cysts were 56.91%, 54.64%, and 44.33%, respectively. Our results are also comparable with El Naggar et al.^23,35^ who recorded the highest percentage of cerebral cyst reduction (53.6%) in infected treated with honeybee venom (BV)-MOF-NPs, followed by the infected groups treated with Ciprofloxacin alone, while the group treated with ciprofloxacin- MOF-NPs, followed by group cured with Spiramycin@MOF-NPs, also group take Bee Venom (BV) alone, then group cured with Spiramycin@MOFs-NPs and lastly group treated with MOF-NPs alone in which the percentages of reduction of brain cysts were (50.4%, 50%, 49.6%, 49%, 48.5%, and 22.8%), respectively. EL-Ashkar et al.^36^ found that the group treated with Spiramycin-metronidazole had fewer brain cysts than the infected control group.

In our experiment, testing the cerebral, eye, and Spleen tissues of mice with chronic toxoplasmosis showed evident effects of MO and Spiramycin when loaded alone on MOFs-NPs. They notably decreased fibrous tissue accumulation, tissue damage, and inflammatory responses. Ginger was proven to have protective properties for the liver, a stimulatory effect on the immune system with cytotoxic activities, a defensive impact against oxidative stress, and antagonizing effects against oncogenic and inflammatory processes^37^. This is in congruence with a study demonstrating the MO@L-AA-Cu palm’s (Moringaolifera) significant alleviation of the inflammatory response^38^. When MO was previously tested in immune suppressed chronically infected mice with cryptosporidiosis, the pathological changes dramatically improved while preserving the intestinal villous pattern. The CO-treated group also showed very mild villous core inflammation, which appeared to be greater than the healing effect of Nitazoxanide^36^.

Parasitological assessment of tissue samples revealed a marked reduction in parasite load in animals treated with MO@L-AA-Cu (G3e), as evidenced by stained tissue smears. This decrease was significant when compared to the parasite burden observed in the positive control group, consistent with previously reported findings^39^. Similarly, the RT-PCR results demonstrated a significant reduction in parasite load in animals treated with MO@L-AA-Cu (G3e) compared to the positive control group (G2), which exhibited the highest gene expression. These results highlight the potential of the herbal-based formulation in effectively suppressing Toxoplasma P29 gene expression following infection. These findings emphasize the therapeutic potential of nanocomposite formulations, especially those integrating *Moringa oleifera* extracts with MOFs, for the treatment of chronic toxoplasmosis. Notably, both MO@L-AA-Cu and Spiramycin@L-AA-Cu treatments resulted in a marked reduction in Toxoplasma P29 gene expression, demonstrating enhanced antiparasitic activity over traditional therapies. The P29 protein, also known as GRA7, is a dense granule protein associated with the parasite’s virulence and its interaction with host cells, further validating its relevance as a therapeutic target^40^. Its gene expression serves as a reliable marker for parasitic load and treatment response. The differential expression of P29 among treatment groups is consistent with prior findings that utilized molecular markers to evaluate T. gondii infection severity^41,42^. The significant efficacy of MO@L-AA-Cu in reducing parasitic load is likely attributed to the synergistic action of *Moringa oleifera* bioactive compounds and the enhanced delivery capabilities of copper-based MOFs. This underscores the promise of combining natural products and nanotechnology to enhance antiparasitic treatments. Spiramycin@L-AA-Cu further demonstrate how nanoformulations can improve drug efficacy and targeting. Continued research should explore underlying mechanisms, evaluate safety, and validate these findings through in vivo studies and clinical trials for chronic toxoplasmosis.

### The synergistic effect of Spiramycin@L-AA-Cu and MO@L-AA-Cu

The remarkable therapeutic efficacy demonstrated by Spiramycin@L-AA-Cu and MO@L-AA-Cu in reducing the brain cyst burden (Table 4) points to a highly synergistic, multi-layered mechanism of action that goes far beyond simple physical drug encapsulation. A major hurdle in treating chronic CNS toxoplasmosis is the restricted passage of conventional therapeutics across the blood-brain barrier (BBB). In our formulation, the use of L-aspartate (L-AA) as the organic linker in the host framework is highly strategic. As an endogenous amino acid, L-AA can actively exploit nutrient transporters highly expressed on the brain capillary endothelial cells, such as the large neutral amino acid transporter 1 (LAT1). This allows the nano-carrier to undergo active, carrier-mediated transport across the BBB, effectively boosting the cerebral bioavailability of both Spiramycin and MO. Once inside the CNS, the framework’s therapeutic cargo is released in a controlled manner. Coordination bonds between Cu^2+^ and the carboxylate groups of L-aspartate are sensitive to local pH. Upon cellular uptake—likely via endocytosis by infected microglia or astrocytes—the acidic microenvironment of endosomes and lysosomes (pH 5.0-6.0) triggers the gradual disassembly of the framework. This prevents a premature burst release of the drug in systemic circulation and ensures a targeted, localized therapeutic concentration inside infected host cells. The structural copper ions (Cu^2+^) released during framework degradation contribute actively to the formulation’s antiparasitic activity. Solubilized Cu^2+^ ions readily penetrate cellular membranes, disrupting the metabolic processes of intracellular tachyzoites and bradyzoites. Copper ions act as catalysts in Fenton-like reactions, leading to the intracellular generation of reactive oxygen species (ROS). This localized oxidative stress damages the structural proteins and lipids of the encysted parasites, explaining why the bare L-AA-Cu framework alone displayed a significant, independent reduction in cyst count (1900 ±32.14).The inclusion of MO in the loaded systems creates a strong therapeutic partnership. Beyond the direct, plant-derived antiparasitic action of active phytochemicals like nitrile glycosides and isothiocyanates, MO exerts potent immunomodulatory and antioxidant properties. The chronic stage of toxoplasmosis is characterized by chronic, low-grade neuroinflammation, where host-driven microglial activation can lead to severe bystander tissue damage. The polyphenolic components of MO (such as quercetin and kaempferol) act as excellent free-radical scavengers. They mitigate the local, copper-induced ROS in healthy host brain tissues, downregulate pro-inflammatory, and preserve the structural integrity of neighboring neurons. Taken together; the L-AA-Cu framework acts not only as a smart, BBB-crossing vehicle but also as an active therapeutic partner. Its pH-responsive release of toxic Cu^2+^ ions works in tandem with the sustained, immunomodulatory, and cysticidal activities of the loaded payloads, leaded to a profound reduction in parasite burden while protecting vulnerable brain tissue.

The therapeutic efficacy of the Cu-MOF@Moringa platform relies on a dual redox mechanism. Polyvalent copper ions (Cu^2+^) embedded within the MOF nodes undergo catalytic redox cycling via a Fenton-like reaction. Concurrently, bioactive phytochemicals present in *Moringa oleifera*—principally quercetin, chlorogenic acid, and kaempferol—act as electron donors. These polyphenols accelerate the reduction of Cu^2+^ back to Cu^+^, maintaining high catalytic turnover for hydroxyl radical (^.^OH) production at pathological sites. Paradoxically, in healthy tissue experiencing oxidative stress, low doses of *Moringa* flavonoids scavenging ambient intracellular ROS protect non-target cells, creating a site-specific, tuned therapeutic window.

### Immune regulation & inflammatory cascade suppression

Beyond direct oxidative modulation, the combined system exhibits immunomodulatory potential. *Moringa* phytochemicals (notably isothiocyanates and quercetin) suppress the activation of Nuclear Factor kappa B, reducing downstream transcription of pro-inflammatory cytokines. Simultaneously, sustained low-level release of Cu^2+^ ions stimulates tissue regeneration and angiogenesis via upregulating Vascular Endothelial Growth Factor (VEGF) and hypoxia-inducible factor 1-alpha. This dual action promotes the repolarization of pro-inflammatory M1 macrophages/microglia toward the regenerative M2 phenotype, dampening chronic inflammation while fostering tissue repair.

### Membrane interaction & blood-brain barrier (BBB) penetration

The enhanced cellular uptake and potential blood-brain barrier (BBB) transit of the hybrid system stem from synergistic physical and chemical characteristics. The small particle size and positive surface of MOFs favor receptor-independent endocytosis (clathrin/caveolae-mediated) over rapid phagocytic clearance. Polyphenolic components from *Moringa* adsorb onto the MOF surface, increasing lipid solubility and affinity for membrane endothelial layers. Localized, controlled micro-ROS generation temporarily destabilizes tight-junction proteins in microvascular endothelial cells, facilitating paracellular and transcellular transport across dense physiological barriers.

### Coordination chemistry & sustained release kinetics

The sustained release profile of *Moringa* bioactive molecules is governed by coordination interactions with open copper coordination sites, as well as hydrogen bonding within the porous framework. In systemic circulation (pH 7.4), the framework remains stable, minimizing premature payload leakage. However, under acidic pathological conditions, protonation of organic linkers destabilizes the metal-ligand coordination bonds. This pH-triggered dissociation ensures a controlled, multi-stage release mechanism: an initial rapid release of surface-bound phytochemicals followed by sustained zero-order diffusion from the inner porous matrix.

### Biosafety considerations and translational risk assessment

While initial *in vitro* cytotoxicity assays in healthy fibroblast cell lines demonstrated low intrinsic toxicity and favorable biocompatibility at therapeutic dose ranges, comprehensive safety profiling for copper-containing nanomaterials requires careful consideration of systemic pharmacokinetics and organ retention. Copper is an essential trace element integral to enzyme catalytic centers; however, unscheduled systemic release of Cu^2+^ can induce oxidative stress via Fenton-like generation of hydroxyl radicals, leading to potential hepatobiliary or renal toxicity. The bioaccumulation risk of the MO@L-AA-Cu system is mitigated through its specific chemical architecture and physiological degradation mechanisms. The inclusion of L-ascorbic acid (L-AA) and nutrient-derived coordination linkers promotes controlled, localized framework breakdown. Once liberated, free Cu^2+^ ions at physiological concentrations are bound by endogenous transport proteins—primarily ceruloplasmin and serum albumin—and safely routed through hepatic storage pathways or cellular efflux mechanisms mediated by ATP7A/ATP7B copper-transporting ATPases. Acid-responsive degradation in endobronchial or lysosomal microenvironments breaks the framework down into low-molecular-weight organic linkers and copper complexes. Sub-nanometer linker species undergo rapid renal filtration, while hepatic processing manages larger particulate degradation fragments for biliary excretion.

### Limitations and future perspectives

While Spiramycin@L-AA-Cu and MO@L-AA-Cu significantly reduced *T. gondii* brain cyst burden, several limitations must be addressed. First, this evaluation was restricted to a single rodent model; future validation in alternative models or human *in vitro* blood-brain barrier (BBB) systems is required. Second, the lack of pharmacokinetic (PK/PD) profiling leaves the exact cerebral bioaccumulation and clearance kinetics of the drugs unquantified. Third, biodistribution studies (using ICP-MS) are necessary to map the clearance organs of the copper-based carrier. Finally, our safety assessment was limited to preliminary *in vitro* cytotoxicity; comprehensive *in vivo* toxicological evaluations—including organ-specific histopathology and liver/kidney biochemical panels—remain essential. Addressing these milestones will be the focus of our next research phase to facilitate clinical translation.

## Conclusion

The findings of this study demonstrate that the integration of *Moringa oleifera* extract and Spiramycin into copper-based metal-organic frameworks (L-AA-Cu) significantly enhances their therapeutic efficacy against chronic toxoplasmosis. The synthesized nanocarriers (MO@L-AA-Cu) achieved a superior reduction in cerebral parasite burden, with the most effective treatment group (G3e) exhibiting a 73.61% decrease in brain cyst count compared to the untreated infected controls. Molecular validation via RT-PCR confirmed these results, showing a marked decline in *T. gondii* DNA levels, while histopathological analysis revealed that the MOF-based delivery system effectively attenuated the inflammatory response, fibrosis, and structural damage within the brain, eyes, and spleen. The enhanced performance of these formulations is likely attributed to the high loading capacity and improved bioavailability afforded by the MOF architecture, which may facilitate the delivery of bioactive compounds across biological barriers. In conclusion, this research highlights the potential of MO-loaded MOFs as a potent, synergistic nanotherapeutic platform. These results provide a compelling basis for further investigation into the pharmacokinetic profiles and long-term safety of MOF-based delivery systems, offering a promising alternative to conventional anti-toxoplasmic therapies that are often limited by toxicity and low cyst-clearance rates.

## Data Availability

The datasets used and/or analyzed during the current study are available from the corresponding author on reasonable request.
